# Multialternative Decision by Sampling: A Model of Decision Making Constrained by Process Data

**DOI:** 10.1037/rev0000102

**Published:** 2018-07

**Authors:** Takao Noguchi, Neil Stewart

**Affiliations:** 1 Department of Experimental Psychology, University College London; 2 Warwick Business School, University of Warwick

**Keywords:** attraction effect, compromise effect, evidence accumulation, sequential sampling, similarity effect

## Abstract

Sequential sampling of evidence, or evidence accumulation, has been implemented in a variety of models to explain a range of multialternative choice phenomena. But the existing models do not agree on what, exactly, the evidence is that is accumulated. They also do not agree on how this evidence is accumulated. In this article, we use findings from process-tracing studies to constrain the evidence accumulation process. With these constraints, we extend the decision by sampling model and propose the multialternative decision by sampling (MDbS) model. In MDbS, the evidence accumulated is outcomes of pairwise ordinal comparisons between attribute values. MDbS provides a quantitative account of the attraction, compromise, and similarity effects equal to that of other models, and captures a wider range of empirical phenomena than other models.

One overarching idea in decision research is that people accumulate evidence for alternatives over time, with a decision reached when the evidence reaches a decision criterion. This sequential accumulation of evidence has proven effective in explaining neural activity during decision (see, e.g., Gold & Shadlen, 2007, for review) and in capturing the time course of perceptual judgments (see, e.g., Ratcliff & Smith, 2004; Teodorescu & Usher, 2013, for reviews). Evidence accumulation provides a general framework for decisions, where values need to be integrated over time or across attributes. Within the multialternative decision making, many implementations of evidence accumulation have been proposed, as listed in Table 1.[Table-anchor tbl1]

One primary difference between the models concerns what, exactly, the evidence is that is accumulated on each step. In some models, transformed attribute values are accumulated. In other models, differences in (raw or transformed) attribute values are accumulated. Other major differences concern the stochastic fluctuation of attention and the choice of decision criterion, as summarized in Table 1.

The contribution of this paper is to present a new model, which we call multialternative decision by sampling (MDbS). This model is an extension of decision by sampling (DbS; Stewart, Chater, & Brown, 2006). The MDbS model differs from the other sequential sampling models of multialternative choice primarily in that the evidence accumulated is pairwise ordinary comparisons on single attribute dimensions. For example, consider a decision between cars: a Ford, a BMW, and a Nissan. The Ford may have a lower price than the BMW, resulting in one unit of evidence accumulated for the Ford. Then, in the next step, the Ford beats the Nissan on fuel efficiency, resulting in one unit of evidence accumulated for the Ford. These steps continue until one car is sufficiently far ahead in evidence units, whereupon a choice is made.

We have used findings from process tracing studies, in particular those on eye-movements, to provide some constraints on how evidence is accumulated. The MDbS model is guided by three constraints in particular. First, the existing literature shows that, in multialternative decision, people’s attention fluctuates between pairs of alternatives on single attributes at one time (Russo & Leclerc, 1994; Russo & Rosen, 1975). So, in the MDbS model, the evidence accumulated is the outcome of a series of evaluations of pairs of alternatives on single dimensions. The link between the attention fluctuation and decision is reported in process-tracing studies (Noguchi & Stewart, 2014; Stewart, Gächter, Noguchi, & Mullett, 2016). The second constraint is that more similar alternatives receive more attention (Noguchi & Stewart, 2014). So, in the MDbS model, more similar alternatives are more likely to be selected for comparison. Third, the distribution of time taken to make a decision (response time) is generally positively skewed and, toward the end of a decision, people attend more to the alternative which they are going to choose (the gaze cascade effect; Shimojo, Simion, Shimojo, & Scheier, 2003; Simion & Shimojo, 2007). Mullett and Stewart (2016) show, in a series of simulations, that positively skewed response times and the gaze cascade effect are consistent only with a decision criteria based on a relative difference in the evidence for each alternative, rather than the absolute evidence for an alternative. So, in the MDbS model, we use a relative decision criteria.

Having used process data to set what would otherwise be arbitrary assumptions about the evidence accumulation in MDbS, we then seek to explain a different set of phenomena: decisions in multialternative choice. Initially we focus upon the so-called big three context effects: the attraction, compromise, and similarity effects. The effects have driven the development of models of multialternative decision because of their theoretical importance and because of the challenge in producing a simultaneous account of all three. We will show that the MDbS’s quantitative account of the big three context effects is as good as two key competing models which also have closed-form solutions for decision probability: multialternative decision field theory (MDFT; Roe, Busemeyer, & Townsend, 2001) and the multiattribute linear ballistic accumulator model (MLBA; Trueblood, Brown, & Heathcote, 2014). We then broaden our consideration of phenomena using a systematic literature survey, and consider the ability of the MDbS model, and other models, to capture the breadth of phenomena. The MDbS model captures almost all of these phenomena without any further assumptions. To begin, we describe the MDbS model.

## Multialternative Decision by Sampling

### Overview

In the MDbS model, evidence is accumulated from a series of ordinal comparisons of pairs of attribute values. The attribute values are drawn from the current choice and from long-term memories of attribute values encountered previously. For example, in evaluating the price of Car A people may compare the price against prices sampled from other alternatives in a choice set: the price of Car B also on offer. People may also compare the price of Car A against prices sampled from long-term memory: prices of other cars they have seen before. No matter the source of the comparison attribute, if the price of Car A is preferable in the pairwise comparison, one unit of evidence is accumulated toward deciding on Car A. This pairwise comparison is considered ordinal, in the sense that evidence is increased one single unit amount regardless of how large the difference is. These ordinal comparisons of pairs of attribute values are sequentially sampled, and drive the evidence accumulation process until the evidence for one alternative is sufficiently far ahead of the evidence for the other alternatives. Below, we expand on this overview.

### Working Memory

In the original DbS model, and in MDbS, working memory contains the attribute values from the choice set and may also contain attribute values retrieved from long-term memory. All of the attribute values, regardless of their source, are processed in exactly the same way. G. D. A. Brown and Matthews (2011) and Tripp and Brown (2015) have integrated a computational model of memory with decision by sampling, but this complexity is not needed to explain the multialternative decisions in this article. Here, working memory is simply the pool of attribute values the decision maker has in the front of their mind. We will see how context effects caused by the addition or removal of alternatives from the current choice set and context effects caused by exposure to attribute values before the current choice are explained by the same mechanism in MDbS.

### Similarity Dependent Comparison Probability

**Figure 1 fig1:**
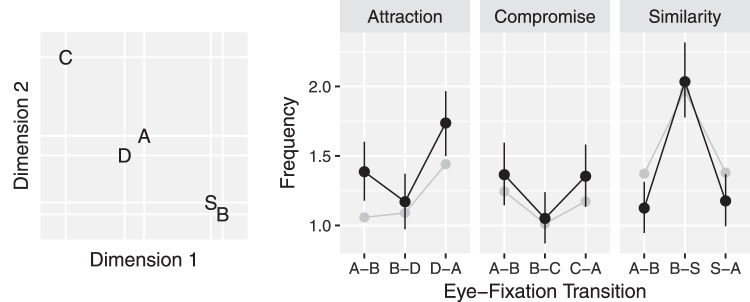
The frequencies of eye-fixation transitions between alternatives for the attraction, compromise, and similarity choices. The labeling of alternatives is shown in the left panel. The second, third, and forth panels show eye-fixation transition frequencies as black dots and MDbS’s predictions of the number of comparisons as gray dots. Error bars are 95% highest posterior density intervals. The frequencies of eye-fixation transitions are redrawn from data reported in Noguchi and Stewart (2014), and the MDbS’s predictions are made with parameter values α = 3, β_0_ = 0.1, β_1_ = 50, and θ = 1.

In the earlier formulation of the DbS model, all attribute value comparisons are equally likely. But process tracing studies suggest that context influences people’s attention. For example, eye-movement studies find that people attend more frequently to alternatives which share attribute values with other alternatives or have similar attribute values (Noguchi & Stewart, 2014; Russo & Rosen, 1975). Figure 1 shows the number of eye-fixation transitions between the three alternatives from an experiment by Noguchi and Stewart (2014) which presented attraction, compromise, and similarity choices, using different cover stories for each choice. The most frequent transitions are between the most similar alternatives. We describe the attraction, similarity, and compromise effects below in detail, but for now note that in the attraction choice set {A, B, D}, transitions are most frequent between pair A and D, prior to a decision. In the compromise choice set {A, B, C}, transitions are most frequent between pair A and B and pair A and C. In the similarity choice set {A, B, S}, transitions are most frequent between pair B and S.[Fig-anchor fig1]

Therefore, in MDbS, the probability of evaluating the value of Alternative *A* on Dimension *i* is proportional to the similarity to the other attribute values in working memory:

$$p(\text{evaluate}\;A_{i})\propto \underset{\overset{X_{i}\in {𝕊}_{i}}{X_{i}\neq A_{i}}}{\sum }\exp\left(-\alpha \;D_{\left(A_{i},X_{i}\right)}\right),$$ 1

where *A*_*i*_ is the attribute value for Alternative *A* on Dimension *i*, ${𝕊}_{i}$ is the set of attribute values from Dimension *i* in working memory, and D is a distance function discussed below.

In MDbS, the probability of evaluating A against B can be different to the probability of evaluating B against A. When averaging over the direction of comparison, MDbS produces the qualitative pattern of comparison frequencies illustrated in Figure 1. The gray dots represent predicted frequencies. In a similarity choice, for example, Equation 1 will assign higher evaluation probabilities to Alternatives B and S than to Alternative A, because B and S both have a high summed similarity to the other alternatives whereas Alternative A does not. Thus, the comparisons which are more frequently made are B against A, B against S, S against A, and S against B. Comparisons of A against B or A against S are less frequent. Because comparisons of B against S and of S against B are both frequent, comparisons *between* B and S are most frequent, as we see in Figure 1.

### Pairwise Ordinal Comparison

In the MDbS model, the rate at which evidence is accumulated for an alternative is determined by two factors: the probability that the alternative is compared on a particular attribute dimension (as described in the previous section), and the probability that the alternative wins the comparison. Formally, the accumulation rate for Alternative A is given by:

$$p(\text{Evidence is accumulated toward }A)=\sum_{i\in 𝔻}p(\text{evaluate}\;A_{i})\;p(A_{i}\text{ wins a comparison})=\sum_{i\in 𝔻}p(\text{evaluate}\;A_{i})\times \left(\sum_{\overset{X_{i}\in {𝕊}_{i}}{X_{i}\neq A_{i}}}p(A_{i}\text{ is compared against }\;X_{i})\;p(A_{i}\text{ is favored over }\;X_{i})\right),$$ 2

where 𝔻 is the set of attribute dimensions along which alternatives are described (e.g., price, comfort and fuel efficiency), and *A*_*i*_ is the attribute value of Alternative *A* on Dimension *i*, and ${𝕊}_{i}$ is the set of attribute values on Dimension *i* in working memory.

The pairwise comparison process is supported by process-tracing studies (e.g., Noguchi & Stewart, 2014; Payne, 1976; Russo & Dosher, 1983; Russo & Leclerc, 1994). These studies show that people move their eyes back and forth between a pair of alternatives on one single attribute value before moving on to the next comparison.

Our assumptions about the ordinality of comparisons—that the evidence accumulation is insensitive to the magnitude of difference between compared values—were grounded in findings from the field of psychophysics, as was the case for the original decision by sampling model (see Stewart et al., 2006). For example, previous research demonstrates that people are rather good at discriminating stimuli (e.g., vertical lines of different lengths, or auditory tones of different loudness) from one another, but rather poor at identifying or estimating the magnitude of the same stimuli (e.g., estimating line length or tone loudness; Laming, 1984; Shiffrin & Nosofsky, 1994; Stewart, Brown, & Chater, 2005), which suggests that ordinal comparisons are relatively easy. In the context of decision making, these studies indicate that people are rather good at judging whether they prefer one attribute value over another, but rather poor at stating exactly how much more they appreciate that attribute value. For example, people are able to clearly state that they prefer the comfort of driving a Mercedes to the comfort of driving a Toyota, but people may not be able to state how much more (e.g., 1.7 times) they prefer the comfort of the Mercedes to the comfort of the Toyota.

### Differences as Fractions

People often behave as if differences are perceived as fractions, as embodied in Weber’s Law. Weber’s Law says that the increment which can be added to a stimulus and just noticed is a constant fraction of the stimulus magnitude. In the context of judgment and decision making, Tversky and Kahneman (1981) report that people are willing to make an extra trip to save $5 on a $15 purchase but unwilling to make the same trip to save $5 on a $125 purchase. This finding suggests that the discount is judged as a fraction and not an absolute value. Although the saving is $5 in both cases, the $5 discount is 33% reduction from the price of $15 but is only 4% reduction from the price of $125. The 4% reduction may not be meaningful enough to influence a decision. Consistent with this finding, changing prices by a small fraction often has only a very small impact on sales (Kalwani & Yim, 1992; Kalyanaram & Little, 1994). Also, studies on employees’ judgments of salary increases find that the increment expressed in a fraction is a better predictor of employees’ judgments of meaningfulness of the increment (Futrell & Varadarajan, 1985; Heneman & Ellis, 1982) and also employees’ subsequent spending and saving decisions (Rambo & Pinto, 1989).

Thus, in MDbS, the distance between *A*_*i*_ and *X*_*i*_ is defined as a fraction:

$$D_{\left(A_{i},X_{i}\right)}=\frac{\left|A_{i}-X_{i}\right|}{\left|X_{i}\right|}.$$ 3

Although this form will behave pathologically when *X*_*i*_ approaches zero, it is sufficient for our purposes. This distance function is used above in Equation 1 for the probability that an attribute value is selected for comparison. It is also used in Equation 4 below for for the probability that an attribute value wins a comparison.

### Probability of Winning a Comparison

The probability that the selected attribute value wins a comparison (i.e., is favored over another value) is given by

$$p(A_{i}\text{ is favored over}\;X_{i})=\{\begin{array}{ll} F\left(\beta_{1}\left(D_{\left(A_{i},X_{i}\right)}-\beta_{0}\right)\right) & \;\text{ if }A_{i}>X_{i}\\ 0 & \;\text{ otherwise}, \end{array}$$ 4

where F is a logistic sigmoid function with β_0_ = 0.1 and β_1_ = 50 in the simulations below. These parameter values mean that an advantage of 10% is favored with .50 probability, and that an advantage of 20% is favored with > .99 probability. Our choice of β_0_ = 0.1 is based on the previous theoretical preposition that people are more sensitive to a difference greater than 10% (Brandstätter, Gigerenzer, & Hertwig, 2006). In using the logistic function, we are replacing the hard comparison between attribute values in the original DbS model with a softer comparison.

To illustrate the softer comparison, suppose we have two identical attribute values and gradually increase one of them. As the difference between the two values grows, it becomes more likely for the larger value to be favored with the soft comparison. This gradual increase in the probability of favoring the value is not possible with the hard comparison, where a small difference is completely ignored and the larger value suddenly becomes favored when the difference grows sufficiently large. We note, however, that Equation 4 can emulate the hard comparison with extremely large β_1_.

Thus far we have defined all of the terms in Equation 2. That is, we have defined what, exactly, the evidence is that is accumulated in MDbS. More detailed walk-throughs of the numerical computation are provided in Appendixes B and C. What remains is to define the stopping rule.

### A Relative Stopping Rule, and a Closed-Form Solution for Decision Probabilities

In models of evidence accumulation, a decision is reached when accumulated evidence satisfies a decision criterion. Empirical evidence suggests that stopping is based upon one accumulator being sufficiently far head of the others (i.e., a relative stopping rule) rather than when the highest accumulator hits a fixed threshold (i.e., an absolute stopping rule; Teodorescu & Usher, 2013). In particular, Mullett and Stewart (2016) used a series of simulations to explore the ability of relative and absolute stopping rules to account for two phenomena: (a) the ubiquitous positively skewed distribution of response times and (b) the gaze cascade effect in which eye fixations are biased more and more strongly toward the alternative ultimately chosen in the second or so before a choice (Shimojo et al., 2003; Simion & Shimojo, 2007). The intuition is as follows, and is based upon the assumption of increased evidence accumulation for the alternative being fixated (Krajbich, Armel, & Rangel, 2010; Krajbich & Rangel, 2011). In an absolute stopping model, the final fixation should be toward the chosen alternative, as that is when the accumulated evidence for that alternative hits the threshold. But the earlier fixations can be in any order. They could be a run of fixations to the nonchosen alternative and then a run to ultimately chosen alternative which catches up, overtakes, and then hits the absolute threshold. Or alternation between the nonchosen and ultimately chosen alternatives, so that the alternatives are neck and neck until the very last fixation. Or many other patterns. But a relative stopping model, where stopping occurs when the evidence for one alternative gets sufficiently far ahead of the evidence for the other, requires a run of fixations to the ultimately chosen alternative. Only a run of fixations produces the gradually emerging gaze cascade effect—the gaze cascade effect is thus a unique empirical signature of a relative stopping rule. Mullett and Stewart (2016) demonstrate this intuition with a comprehensive series of simulations. (Note, also, that the argument is separate from the issue of whether there is a causal effect from accumulated evidence to fixations—it is not required that people are more likely to look at the thing they prefer.) In summary, only a relative stopping rule is consistent with the process tracing evidence, and so, in MDbS, we assume a relative stopping rule (see also, Nosofsky & Palmeri, 1997).

For a decision between more than two alternatives, the criterion is likely to be either (a) a difference between the maximum and next-best evidence or (b) a difference between a maximum and a mean-average evidence (for discussion, see Teodorescu & Usher, 2013). Further experimental work is required to discriminate between these possibilities. Here, for computational feasibility, we assume that a decision is made when a difference between a maximum and a mean-average evidence reaches a threshold θ = 0.1. This threshold value means that, on average, 2.5 comparisons are made prior to a decision in attraction, compromise, similarity choices. By conceptualizing the evidence accumulation as a random walk over accumulator states, we have been able to follow Diederich and Busemeyer (2003) and develop a closed form solution for the decision probabilities. Appendix A gives the derivation.

## Testing DbS Mechanisms

In this section, we discuss earlier studies which tested the DbS mechanisms. This work focused upon the predictions the DbS model makes when the attribute values in working memory are manipulated.

### Incidental Value Effect

In the DbS and MDbS models, the attribute values that happen to be in working memory determine how much a given attribute value contributes to accumulation rates. Suppose that a decision maker happens to have £1, £2, and £7 in working memory. A target value of £5 will win in each pairwise comparison against £1 and £2, but will lose the comparison against £7 (assuming these differences are sufficiently large). Thus the target £5 will win in two out of three comparisons. Then the probability that the £5 alternative wins a comparison is 2/3 = .67.

More generally, the probability that an attribute value wins a comparison is closely related to its relative rank within values in working memory. A relative rank is the proportion of attribute values to which a target value compares favorably. In the above example, the relative rank of £5 is .67. When a relative rank is high, an attribute value is more likely—by definition—to win a comparison, leading to a higher accumulation rate and ultimately contributing to a higher decision probability for the alternative.

This predicted relation between a relative rank and decision was tested by Ungemach, Stewart, and Reimers (2011), who offered a decision between two probabilistic pay-offs to consumers as they left a supermarket. One alternative offered a .55 probability of £0.50 and otherwise nothing; and the other offered a .15 probability of £1.50 and otherwise nothing. Ungemach et al. (2011) used the supermarket receipt as a proxy for the values that the customer had recently experienced and would likely be in his or her working memory. Ungemach et al.’s (2011) results show that the more values on the receipt that fell between the £0.50 and £1.50 prizes, the more likely that the lottery for £1.50 was chosen. According to DbS, this is because when more prices fall between the £0.50 and £1.50 prizes, the relative rank of these prizes differs more. Of course, the supermarket prices experienced should not have affected the lottery decision, but, according to the DbS and MDbS models, because these values remained in working memory at the time of the lottery decision, they affected the relative ranks of £0.50 and £1.50, and thus affected the lottery decision.

### Attribute Distribution Effect

When people are faced with a series of questions, the attribute values from earlier questions can remain in working memory and affect subsequent decisions. Thus different distributions of attribute values in earlier questions should have a systematic effect on subsequent decisions. We illustrate this with an example from Stewart, Reimers, and Harris (2015).

Stewart et al. (2015) compared two distributions. In the first, monetary rewards in working memory were positively skewed, with values £0, £10, £20, £50, £100, £200, and £500. In the second, the values were uniformly distributed, with values £0, £100, £200, £300, £400, and £500. Consider one of the attribute values common to both distributions, say £200. In the positively skewed distribution, it has a relative rank of 5/7 = .71 because it compares favorably to five of the seven attribute values (£0, £10, £20, £50, and £100). In the uniform distribution, it has a relative rank of 2/6 = .33 because it compares favorably with only two out of the six attribute values (£0 and £100).

**Figure 2 fig2:**
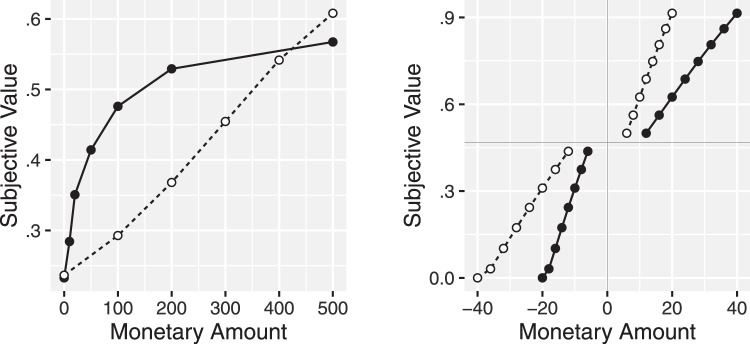
Multialternative decision by sampling (MDbS) predictions for the attribute distribution effect (the left panel) and the loss aversion effect (the right panel). Accumulation rates for a particular attribute value are mean-averaged over all possible comparisons with other available values to derive the MDbS subjective value. In both panels, dots represent the monetary pay-offs presented during the experiment, and a line connects all of the amounts available within a condition.

Figure 2 plots the subjective value functions for money for these positively skewed and uniform distribution conditions. These subjective values are computed as the average accumulation rate for the target attribute value (Equation 2). The general principle is that the probability that a target attribute value wins a comparison increases most quickly, and thus a subjective value increases most quickly, in the most dense parts of the attribute value distribution. (Note that the slight deviation from linear for the uniform distribution condition and there is also slight variation in the positive skew condition which is harder to see. This is caused by the effects of similarity on the rate at which targets are selected for comparison, as is the crossing of the lines near £400–£500.)[Fig-anchor fig2]

To test this prediction, Stewart et al. (2015) asked participants to make a series of risky decisions between probabilistic pay-offs. Different participants had their pay-off amounts drawn from different distributions. Just as the MDbS model predicts, the estimated value showed greater concavity when the distribution is positively skewed than when it is uniformly distributed (or in other experiments, negatively skewed). In the traditional expected utility framework, the concavity of the subjective value (or utility) function indexes the level of risk aversion displayed. The profound implication of Stewart et al.’s (2015) result is that the level of risk aversion is a property of the questions the experimenter asks, and not on the people making the decisions—at least to a large extent.

The question remains as to why then we typically see risk averse, concave subjective value functions in most laboratory experiments and in estimations from real-world decisions. Stewart et al. (2006) observed that the distribution of attribute values is very often positively skewed in a number of domains, including credits and debits to bank accounts and supermarket prices (see also, Stewart & Simpson, 2008; Stewart, 2009). If the real-world has mostly positively skewed distributions, we should see mostly risk averse, concave subjective value functions. Stewart et al. (2006) also made similar observations linking the distribution of probabilities in the world to inverse-S-shaped weighting functions and the distribution of delays in the world to hyperbolic-like discounting functions. Changes in the distributions of attribute value can also explain key phenomena in risky decision (e.g., the common ratio effect, Stewart & Simpson, 2008; Stewart, 2009).

### Loss Aversion

According to the DbS and MDbS models, the distribution of values in working memory offers an explanation of loss aversion. People often behave as if losses loom larger than gains (see Camerer, 2005; Fox & Poldrack, 2014, for reviews). For example, when offered a decision to play gambles with equal chance to win or lose an amount people typically reject such an offer (also see Gal, 2006). Famously, loss aversion was incorporated into the subjective value function in prospect theory (Kahneman & Tversky, 1979), which shows a steeper curve in the loss domain than in the gain domain.

In the DbS and MDbS models, loss aversion is explained through an asymmetry in the ranges of the distributions of gains and losses typically used in measuring loss aversion. For example, suppose gains are drawn from a uniform distribution between £0 and £40, but losses are drawn from a uniform distribution between £0 and £20 (e.g., after Tom, Fox, Trepel, & Poldrack, 2007). An increase from £0 to £10 covers one half of the values in the £0–£20 distribution of losses but only one quarter of the values in the £0–£40 distribution of gains. Thus the same amount should perceived as larger in losses than gains: people should be more sensitive to losses. When the distributions are reversed so that losses are drawn from a uniform distribution between £0 and £40 and gains are drawn from a uniform distribution between £0 and £20, this sensitivity should also be reversed.

The right panel of Figure 2 shows the predicted subjective value function from the MDbS model, which shows exactly this pattern. These predictions were tested by Walasek and Stewart (2015), who showed the usual loss aversion when the range of losses was narrower than the range of gains. When gains and losses were symmetrically distributed weak or zero loss aversion was observed, and when the distributions were reversed the opposite of loss aversion was observed.

The three phenomena we have reviewed above were designed to test predictions from the DbS model and were run by Stewart and colleagues. Below, we review the empirical findings which were not designed to test the DbS model, and discuss how the MDbS model explains the findings.

## The Big Three Context Effects: Attraction, Compromise, and Similarity

The attraction, compromise, and similarity effects are central to the psychology of multialternative decision because of their theoretical importance—their very existence rules out the most obvious accounts of how people make decisions. For example, an obvious class of model, foundational in normative economic models of multialternative decision, is the class of simple scalable models. A model has the property of simple scalability if the value of each alternative can be represented by a single scalar (a single real number), with the probability of choosing an alternative increasing in its value and decreasing in the value of other alternatives (see Roe et al., 2001, for a review).

A classic simple scalable model is Luce’s choice rule, where the decision probability for Alternative A is given by $p(A|\{A,\;B,\;C\})=\frac{V_{A}}{V_{A}+V_{B}+V_{C}}$ where the *V*_*X*_s are the values for each of the *X* = {*A*, *B*, *C*} alternatives. The scalable models have the property that adding an alternative to a choice set cannot reverse the ordering of the decision probabilities for existing alternatives. For example, if *p*(*A*|{*A*, *B*}) > *p*(*B*|{*A*, *B*}) then *p*(*A*|{*A*, *B*, *C*}) > *p*(*B*|{*A*, *B*, *C*}). This property is called independence from irrelevant alternatives, and follows for the Luce model, for example, because if $p(A|\left\{A,\;B\right\})=\frac{V_{A}}{V_{A}+V_{B}}>\frac{V_{B}}{V_{A}+V_{B}}=p(B|\left\{A,\;B\right\})$ then *V*_*A*_ > *V*_*B*_, which means $p(A|\left\{A,\;B,\;C\right\})=\frac{V_{A}}{V_{A}+V_{B}+V_{C}}>\frac{V_{B}}{V_{A}+V_{B}+V_{C}}=p(B|\left\{A,\;B,\;C\right\})$. In a stricter form, Luce’s choice axiom states that the ratio $\frac{p(A|𝕊)}{p(B|𝕊)}$ is constant and independent of 𝕊.

These scalable models also imply that adding an alternative to a choice set cannot increase the decision probability for any existing alternative: $\frac{V_{A}}{V_{A}+V_{B}+V_{C}}$ cannot be greater than $\frac{V_{A}}{V_{A}+V_{B}}$ for any positive values of *V*_*A*_, *V*_*B*_, and *V*_*C*_. This property is called regularity. The properties of independence from irrelevant alternatives and regularity are not compatible with the big three context effects: as we discuss below, the big three context effects demonstrate that people can reverse their relative preference for two alternatives and can become more likely to choose an existing alternative after a new alternative is introduced. Thus the existence of the big three context effects rules out the Luce choice model and all other simple scalable models, such as the Thurstonian model (Thurstone, 1927) and multinomial logistic regression.

**Figure 3 fig3:**
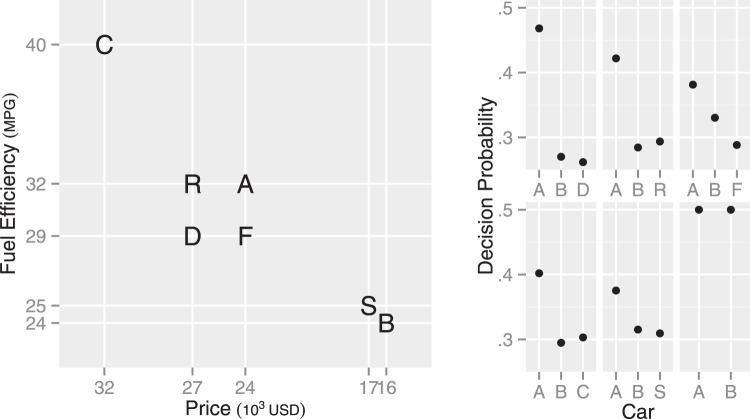
Cars to illustrate the big three contest effects (left panel) and the demonstration of the effects with the multialternative decision by sampling (MDbS) model (the six right panels). The big three context effects document that decision probabilities of Car A and B depend on the presence or absence of Car D, R, F, C, or S in a choice set. The six right panels show that the decision probabilities predicted by the MDbS model: the decision probability for Car A is higher than other cars with presence of Car D (top left), Car R (top middle), Car F (top right), Car C (bottom left), and Car S (bottom middle). When only Cars A and B are in a choice set, the decision probability for Car A is the same as that for Car B (bottom right).

In discussing the big three context effects below, we use an example decision between cars illustrated in the left panel in Figure 3. Available cars are described in terms of the two attributes, price (in U.S. dollars) and fuel efficiency (in miles per gallon). Here, Car A is better than Car B on the fuel efficiency but Car B is better than Car A on the price (see Figure 3). Attribute values were selected from the U.S. car market in May, 2015, such that the MDbS model predicts indifference between Cars A and B when only Cars A and B are in a choice set.[Fig-anchor fig3]

In simulating the big three with the MDbS model, we use a fixed, single set of parameter values throughout this article. We set the similarity parameter α = 3.0, the soft ordinal comparison parameters for the logistic β_0_ = 0.1 and β_1_ = 50, and the decision threshold θ = 0.1, as described above. For brevity of explanation, we assume that people are unfamiliar with the choice domain and cannot sample values from long-term memory beyond those in the immediate choice set. The significance of this assumption is addressed when we discuss how familiarity with choice domain affects the strengths of the context effects.

### The Attraction Effect

To illustrate the attraction effect, suppose a choice set contains Cars A, B, and D. Car D is inferior to Car A in both attributes (see the left panel in Figure 3). Thus Car D should be discarded from consideration but, after adding Car D to the choice set, Car A becomes more likely to be chosen than Car B (Huber, Payne, & Puto, 1982). Adding Car D often increases the choice share for Car A, which is a violation of regularity.

While noting that several explanations are possible, Huber et al. (1982) primarily discussed the attraction effect in terms of shifts in weights: addition of Car D would lead people to weight fuel efficiency more heavily as this is where Car D (and also Car A) is advantageous (Huber et al., 1982; see also Bhatia, 2013). This weight-shift account has received mixed support from subsequent studies (e.g., Wedell, 1991).

In the MDbS model, however, the attraction effect is explained with changes in the probability of winning comparisons when Car D is added. Table 2 has 12 rows that correspond to all of the possible pairwise comparisons in the attraction effect choice set (three cars can be target × two cars can be comparisons for each target × two dimensions). The addition of Car D in a choice set increases the probability that Car A wins attribute value comparisons, because Car A compares favorably with Car D on both price and fuel efficiency, whereas Car B only compares favorably on price. Also, as Cars A and D are similar, they are selected as targets for comparison more often, as the bold in Table 2 indicates. This amplifies the effect Car D has on Car A. It also increases the selection of Car D as target, but as Car D has so few possible favorable comparisons, Car D has the lowest rate of evidence accumulation. The right panel of Figure 3 shows the predictions for decision proportions, with Car A having a higher probability of being chosen when Car D is added to the choice set. What Table 2 is illustrating is the balance between the changes in the favorable comparisons when Car D is added and changes in the attention each car receives when Car D is added.[Table-anchor tbl2]

#### Location of the Decoy

Previous research reports that the strength of the attraction effect can depend on the location of the decoy car (Huber et al., 1982; Wedell, 1991): Car A is more likely chosen when a choice set contains Car R than Car F (see the left panel in Figure 3 for the attribute values of each car). As a potential explanation, Huber et al. (1982) suggest that the advantage of Car B over Car A on price may be perceived smaller with the presence of Car R, as the presence of Car R widens the range of prices in the choice set.

The MDbS model’s explanation is in line with Huber et al. (1982)’s suggestion. By widening the range of prices, the presence of Car R increases the probability that Car A is favored in a comparison on price. In addition, compared with Car *F*, Car R is further away from Car B, and thus, Car B is less frequently evaluated when Car R is in a choice set than when Car F is. The infrequent evaluation of Car B means more frequent evaluation of Car A. Overall, Car A is more frequently evaluated when Car R is in the choice set than when Car F is. As a result, Car A has a higher decision probability with Car R than Car F (the right panels in Figure 3), explaining the varying strength of the attraction effect.

#### Distance to the Decoy

**Figure 4 fig4:**
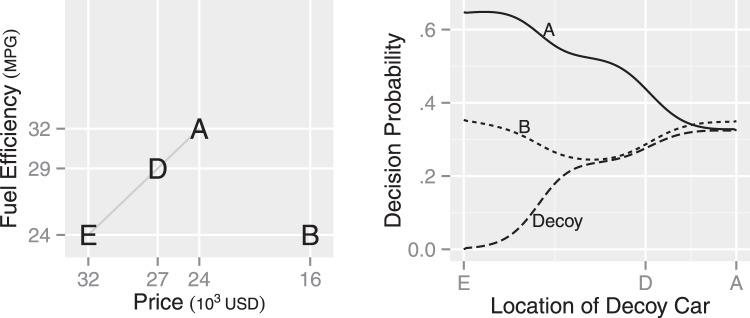
Effects of varying distance between Car A and the decoy car. The decoy car is located along the gray line in the left panel. The right panel plots the decision probability for each car as a function of the decoy car’s location. The attraction effect briefly strengthens as the decoy car moves away from Car E, but gradually weakens as the decoy car moves closer to Car A.

The attraction effect is also reported to be weaker when the decoy car, which is inferior to Car A, is more similar to Car A (Soltani, De Martino, & Camerer, 2012). To explore this finding with the MDbS model, we move the decoy car along the gray line in Figure 4, from Car E, through Car D, to Car A. As the decoy car comes closer to Car A, the decoy car gradually appears better on the fuel efficiency than Car B. As a result, the decision probability for the decoy car initially increases. As the decoy car becomes very similar to Car A, the advantage of Car A over the decoy becomes less likely to be recognized because of the soft threshold for winning comparisons. Thus, the decision probability for Car A gradually decreases, and the attraction effect eventually diminishes.[Fig-anchor fig4]

### The Compromise Effect

The addition of Car C to the choice set with Cars A and B produces the compromise effect (see the left panel in Figure 3 for the attribute values of each car). Car C has extremely good fuel efficiency but comes with very high price. Importantly, Car C makes Car A a compromise between the other cars and, with Car C’s presence, Car A becomes more likely to be chosen than Car B (Simonson, 1989).

This compromise effect has been associated with difficulty in making a decision (Simonson, 1989): as people are uncertain about which attribute dimension is more important, people find a decision on the compromise alternative (Car A) easiest to justify and hence they are more likely to decide on Car A.

The MDbS model’s explanation is quite different. When a choice set contains Cars A, B, and C, Car A is most frequently evaluated. This is because Car A is most similar to other cars. Table 2 shows that, although each car wins two of the four possible comparisons, Car A is most frequently evaluated as a target, leading to a higher decision probability for Car A than for Car B or C (see the right panels in Figure 3). This higher frequency of evaluation of more similar pairs is seen very clearly in the eye tracking data from Noguchi and Stewart (2014), as shown in Figure 1.

### The Similarity Effect

In the similarity effect, introducing Car S to a choice between Cars A and B (see the left panel in Figure 3) robs decision probability more from the similar Car B than the dissimilar Car A (Tversky, 1972). This similarity effect was first explained with elimination by aspects (Tversky, 1972), in which one attribute dimension is attended at one moment, and all of the alternatives which do not meet a certain criterion on the dimension are eliminated from consideration. When alternatives are similar, they tend to be eliminated together or remain together. The elimination process continues until all alternatives but one are eliminated. In the choice set of Cars A, B, and S, people may attend to fuel efficiency at one moment, judge Cars B and S to be poor, and eliminate Cars B and S from consideration leaving Car A to win. If people attend to price, however, Car A will be eliminated leaving Cars B and S in consideration and ultimately to share victory. Thus probability that Car A is chosen will be higher, because when it remains it remains on its own and does not end up sharing a victory.

In contrast, the MDbS model explains the similarity effect with people’s tendency to ignore relatively small differences. Specifically, the differences between Cars B and S are so small that the differences are not very likely to be recognized. Table 2 shows that although Cars B and S are similar to each other and hence are more frequently evaluated, the small differences reduce the probability that Cars B and S are favored in pairwise comparisons. Consequently, the decision probabilities for Cars B and S are lower than the decision probability for Car A (the right panels in Figure 3).

### Familiarity With the Choice Domain

**Figure 5 fig5:**
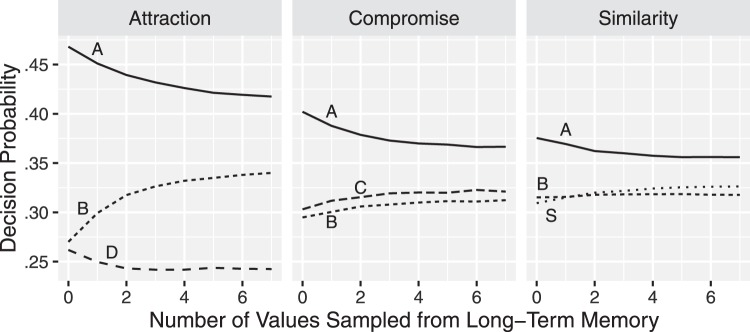
Decision probability as a function of the number of attribute values sampled from long-term memory. Each panel summarizes mean-average decision probability of 5,000 simulations for each number of samples. With the number of samples from long-term memory, the attraction (left panel), compromise (middle panel), and similarity (right panel) effects all become weaker. In this illustration, attribute values in long-term memory are assumed to follow normal distribution whose mean is the attribute values of Car A, and standard deviation is the absolute difference between Cars A and B. We also assumed that attribute values in long-term memory are weakly correlated at Pearson coefficient = −.2. However, the findings of weakened effects with more long-term memory samples are robust across many possible distributions.

Familiarity with the choice domain reduces strength of the attraction effect (Kim & Hasher, 2005) and the compromise effect (Sheng, Parker, & Nakamoto, 2005). In our application of MDbS model above, the attraction, similarity, and compromise effects emerge purely from the comparisons within the attribute values from the choice set. But in addressing the Ungemach et al.’s (2011) supermarket experiment, Stewart et al.’s (2015) attribute distribution effects, and Walasek and Stewart’s (2015) malleability of loss aversion the effects emerge from the comparison with attribute values from earlier choices, which we assume remain in working memory, or are recalled from long-term memory. The effects of familiarity are also attributed to the sampling of attribute values from long-term memory. It seems quite reasonable to assume that those unfamiliar with the choice domain will have few values to sample from long-term memory, and that experience will provide more values to sample. As more values are sampled from long-term memory, they dilute the effect of the comparisons within the immediate context that were driving the big-three effects, reducing their strength, consistent with the effects of familiarity. To demonstrate, we modeled the attribute values in long-term memory with multivariate normal distribution and examined how decision probability changes as more samples are drawn from long-term memory. The results are summarized in Figure 5: As the number of values sampled from long-term memory increases, the big three context effects become weaker.[Fig-anchor fig5]

### Time Pressure

**Figure 6 fig6:**
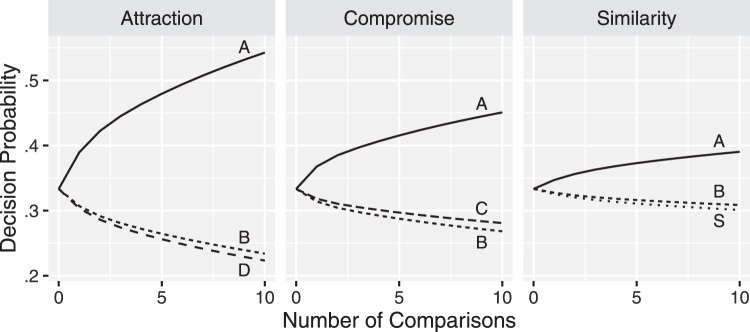
Decision probability for each car after a fixed number of comparisons. The three panels show that as the number of comparisons increases (i.e., time pressure is reduced), the attraction (the left panel), compromise (the middle panel), and similarity (the right panel) effects become stronger.

Previous studies report that the attraction, compromise, and similarity effects are weaker when a decision is made under time pressure (Pettibone, 2012; Trueblood et al., 2014). This is because under time pressure, people may not have enough time to evaluate each alternative, and a decision tends to be more random (Pettibone, 2012). We implement this time pressure effect in the MDbS model by limiting the number of pairwise ordinal comparisons made to reach a decision. Figure 6 reports these simulations. In the simulations, when two or more cars accumulate the same strength of evidence, one car is randomly chosen. When fewer comparisons are made, decisions are made with less evidence and the big-three effects diminish in size.[Fig-anchor fig6]

### Correlations Between the Strengths of the Big Three Context Effects

Since the specification and initial submission of the MDbS model, we have applied it, unchanged, to new evidence about the correlations across individuals of the strengths of the big-three context effects. In a meta analysis, Tsetsos, Scheibehenne, Berkowitsch, Rieskamp, and Mata (2017) show the size of the attraction and compromise effects are positively correlated, and that both are negatively correlated with the size of the similarity effect.

**Figure 7 fig7:**
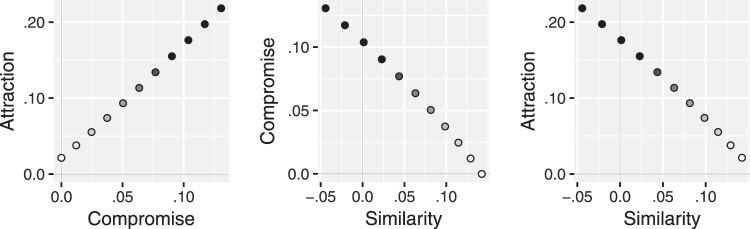
Correlations between the strengths of the attraction, compromise, and similarity effects over individuals. A larger value indicates that the effect is stronger, a value of zero indicates that the effect is not predicted, and a negative value indicates that the effect is reversed. The value of α is indicated with the interior color of dots: white color represents the prediction with α = 0, and black color represents the predictions with α = 5.

These correlations can be explained with variety of mechanisms (Tsetsos et al., 2017). In the MDbS model, one possible way to capture these correlations is via individual differences in the similarity parameter α. To demonstrate, we followed Tsetsos et al. (2017) and computed the relative choice share of Alternative A over B while varying the similarity parameter α from 0 to 5. The MDbS predictions are illustrated in Figure 7, which shows that as the similarity parameter becomes large, the attraction and compromise effects become stronger, but the similarity effect becomes weaker—mimicking the correlations seen in the meta analysis.[Fig-anchor fig7]

### A Quantitative Comparison of Closed-Form Models of the Big Three Context Effects

We have demonstrated that the MDbS model can produce a qualitative account of the big three context effects using one fixed specification of the model with one fixed set of parameter values. In this section we offer a quantitative evaluation of the predictive accuracy of the MDbS model. We use data from a new experiment where participants chose between consumer goods and simultaneously show all of the big three context effects. We compare the predictive accuracy with those of MDFT and MLBA models, two other models designed to capture the big three context effects that also have closed form solutions.

#### Multialternative Decision Field Theory (MDFT)

Decision field theory (Busemeyer & Townsend, 1993) was originally developed to explain decisions between two alternatives but was later extended to explain decisions between more than two alternatives (Roe et al., 2001). MDFT was the first simultaneous account of the attraction, compromise, and similarity effects.

In MDFT, on each time step in the accumulation process, attention is focused on one dimension and attribute value differences are accumulated for all alternatives. In the next time step, attention can switch to a new dimension. During the accumulation process, accumulators are subject to distance-dependent lateral inhibition, where evidence accumulated for one alternative inhibits evidence accumulated for another alternative, and the strength of the inhibition depends on distance between two alternatives in attribute space. The computational implementation of MDFT is described in Appendix D.

The explanation of the similarity effect is similar to that of elimination by aspects. The switching of attention across attribute values means that cars similar in attribute space receive correlated inputs for their accumulators. For example, Cars B and S have positive accumulation when price is attended and negative accumulation when fuel efficiency is attended. Car A shows the opposite pattern, having positive accumulation when fuel efficiency is attended and negative accumulation when price is attended. This means that Cars B and S tend to have very similar accumulated evidence at each time point and thus end up competing for and sharing wins when price happens to dominate the sampling of attention. Car A however does not have such competition, and so gets all of the wins when fuel efficiency happens to dominate the sampling of attention.

The explanation of the compromise effect is that the distance-dependent lateral inhibition creates a correlation in accumulated evidence between Cars B and C. The logic is as follows. Because the distant dependent inhibition is stronger between the more similar Cars A and C and Cars A and B, the evidence accumulated for these pairs tends to become anticorrelated. If C and B are both anticorrelated with A, then they will become correlated with one another. This means that Cars C and B end up sharing wins—as when one does well so does the other. But Car A does not have to share wins and thus has an advantage.

Finally, the explanation of the attraction effect also depends on distance-dependent lateral inhibition. As Car D is inferior to Car A in both attributes, attribute value differences tend to be negative for Car D, causing the evidence accumulated for Car D to become negative. This negative accumulation, when propagated through the lateral inhibition, gives a positive boost for Car A’s accumulator. Car B is sufficiently distant from Car D that Car B’s accumulator is unaffected by inhibition from Car D’s negative accumulation and thus Car B does not receive a boost. Thus only Car A and not Car B is boosted by Car D, and so Car A has the highest decision probability.

This explanation of the attraction effects was criticized as neurally implausible because of the reliance upon negative accumulator values (Usher & McClelland, 2004), although neurons can drop below threshold levels of firing. Tsetsos, Usher, and Chater (2010) also criticized the account of the attraction effect, pointing out that introducing an extra decoy D’ dominated by Car D should reverse the effect: The worse decoy Car D’ will eventually develop negative evidence, which should lead to boosted accumulation for Car D, which in turn should inhibit accumulation for Car A, creating a reverse attraction effect. Under this reverse attraction effect, Car B is more likely chosen over Car A. It seems improbable that the addition of another alternative inferior to Car A decreases the probability of Car A being chosen, but this reverse attraction effect has not been empirically examined.

#### The Multiattribute Linear Ballistic Accumulator (MLBA) model

The linear ballistic accumulator model was originally developed as a simplified model of evidence accumulation (Brown & Heathcote, 2008) but later extended to account for the attraction, compromise, and similarity effects as the MLBA model (Trueblood et al., 2014). In the MLBA model, evidence for each alternative is accumulated at a constant but noisy rate. In the MBLA model, the sequential sampling aspect of the accumulation is dropped in favor of a ballistic accumulation process with rates fixed over the duration of the accumulation. The MBLA model also has no assumptions of lateral inhibition between accumulators. Instead, the accumulation rate is determined by sum of weighted advantage of an alternative’s subjective values. The subjective value function in the MLBA model favors an alternative with similar attribute values across dimensions. When attribute values range from 0 to 10 on two dimensions, for example, the sum of subjective values for attribute values (5, 5) is higher than that for attribute values (2, 8). In the weighting of advantages, the weight is distance-dependent: a small difference in subjective values is more heavily weighted than a large difference. Further, a disadvantage is more heavily weighted than an advantage. The computational implementation of the MLBA model is described in Appendix E.

The explanation of the attraction effect is through the distance-dependent weights on advantages. As a small advantage is more heavily weighted than a large advantage, the advantage of Car A over Car D is more heavily weighted than the advantage of Car B over Cars A or D. As a result, Car A has a higher accumulation rate than Car B. This mechanism is analogous to the distance-dependent lateral inhibition in the MDFT model. Just as the lateral inhibition creates a positive boost only to Car A in the MDFT model, only Car A gains from the presence of Car D in the MLBA model.

The explanation of the similarity effect is through the distance-dependent weights on disadvantages. Only Car A has no small disadvantages—only two large disadvantages to Cars B and S on price. In contrast, Car S has one small disadvantage to Car B on price (the disadvantage to Car A on fuel efficiency is large). Similarly Car B has one small disadvantage to Car S on fuel efficiency (the disadvantage to Car A on fuel efficiency is large). As small disadvantages are heavily weighted, Cars S and B are disadvantaged whereas Car A, which has no small disadvantages, is not. Thus Car A has the highest accumulation rate. Therefore just as Cars B and S inhibit each other in the MDFT model through the distance-dependent lateral inhibition, Cars B and S lower the accumulation rates of each other in the MLBA model through the distance-dependent weights on disadvantage.

For the compromise effect, the cars are more distant from one another. The weight for the medium differences between Cars C and A and Cars A and B is similar to the weight the large differences between Cars C and B. The compromise effect is, instead, explained through different weights on advantages and disadvantages. In particular, Car B’s disadvantage to Car C on fuel efficiency is given more weight than Car B’s advantage over other cars on price. As a result, Car B has a small accumulation rate. Similarly, Car C’s disadvantage to Car B on price is heavily weighted, and thus, Car C has a small accumulation rate. On average, Car A has smaller disadvantages over other cars, and as a result, Car A has the largest accumulation rate. In addition, Car A has a higher subjective value than Cars B and C. This is because the subjective value function requires attribute values to be on the same unit and range across dimensions, and when attribute values are on the same unit and range, Car A has similar attribute values for both dimensions.

The above explanation of the big three context effects stands upon a fine balance between the weights. The MLBA model has been criticized as being too sensitive to small changes in attribute values. Tsetsos, Chater, and Usher (2015), in particular, show that for all of the combinations of reasonable parameter values, it is possible to reverse the attraction effect (i.e., to make Car B preferable over Car A) by introducing small changes to the attribute values (see also Trueblood, Brown, & Heathcote, 2015). As we describe above, a reverse attraction effect has not been found.

#### Big three consumer choices experiment

To allow us to compare the MDbS, MDFT, and MLBA models on their ability to capture the big three context effects with consumer choices, we have run a new experiment.

#### Method

We collected data from 503 participants (204 female, 298 male and 1 undisclosed, whose age ranges from 18 to 75 with the mean of 33) recruited through Amazon Mechanical Turk (https://www.mturk.com). Each participant was paid $1.00 for taking part.

We asked each participant to make eight decisions in a random order: two control decisions between three alternatives, where one alternative dominates the other two; three decisions between three alternatives, each of which was intended to invoke the attraction, compromise and similarity effects; and three decisions between two alternatives. Each alternative was described in terms of two dimensions. Two or three alternatives were displayed in a table, and the participants made decisions by clicking on an alternative. In the table, attribute dimensions were organized in rows, and alternatives were organized in columns. The order of columns (e.g., which alternative to appear on the left column) and the order of dimensions (e.g., which dimension to appear on the first row) was randomly shuffled for each participant for each trial.

The decisions in the experiment were between various consumer products. We prepared eight consumer product cover stories (e.g., mouthwash, and boxes of chocolate; see Appendix F for the complete list). Each cover story contained two alternatives, and these alternatives were presented to participants for the two-alternative decisions. For the three-alternative decisions, we randomly selected one of the two alternatives and generated a third alternative in a way that the context favors the selected alternative.

To generate a third alternative for an attraction choice, for example, we first calculated the absolute differences between Alternatives A’s and B’s attribute values on each dimension. To create an attraction choice which favors Alternative A, we generated Alternative *D*_*A*_ by subtracting 25% of the differences from Alternative A’s attribute values, so that Alternative *D*_*A*_ is inferior to Alternative A in both dimensions. To create Alternative *D*_*B*_, we subtracted the same 25% from Alternative B’s attribute values. To generate a third alternative for a similarity choice to favor Alternative A, similarly, we subtracted 2% of the A-B difference from Alternative B’s attribute value on one dimension and added 2% of the A-B difference to Alternative B’s attribute value on the other dimension.

As a result, each cover story has two versions of choice sets for each of attraction, compromise, and similarity effects: one whose context favors Alternative A and the other whose context favors Alternative B.

We decided, in advance of data collection, to recruit 500 participants and remove the data collected from the participants who choose a dominated alternative in either or both of the control choices.

#### Results and modeling

**Figure 8 fig8:**
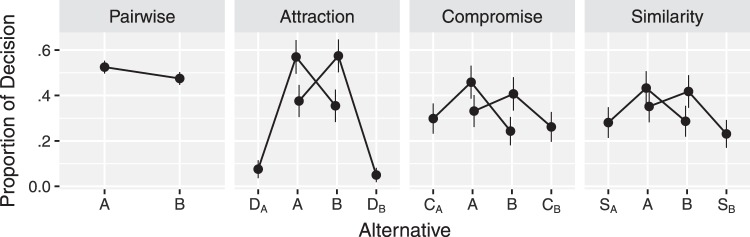
Choice proportions for each alternative in the big-three consumer choices experiment. Each panel represents an experimental condition: the pairwise condition is where only two alternatives (A and B) were presented. The attraction, compromise and similarity conditions are where three alternatives were presented to replicate the attraction, compromise and similarity effects. A solid line connects a choice set, and error bars are 95% confidence intervals.

Of the 503 participants, 150 chose a dominated alternative in one or two control choices, and we removed the data collected from those participants, as we had decided in advance of data collection. The data from the remaining 353 participants are summarized in Figure 8, which shows replication of the attraction, compromise, and similarity effects (see Appendix G for an additional exploratory analysis). The far left panel in Figure 8 shows that, across cover stories, Alternative A was not strongly preferred or disliked over Alternative B. The three right panels in Figure 8 show replications of the attraction, compromise and similarity effects: Alternative A is most often chosen, when the third alternative (*D*_*A*_, *C*_*A*_, or *S*_*A*_) was positioned in a way intended to favor Alternative A under the expectation of replicating the attraction, compromise and similarity effects. In contrast, when the third alternative (*D*_*B*_, *C*_*B*_, or *S*_*B*_) was positioned in a way intended to favor Alternative B, Alternative B was most often chosen.[Fig-anchor fig8]

Although the attribute values in the experiment have different scales and units, the MDFT and MLBA models require attribute values to be on the same scale and unit. Thus for the MDFT and MLBA models, we linearly transformed attribute values, such that Alternative A always had attribute values (3, 2) and Alternative B had attribute values (2, 3).

In fitting the models, we used a hierarchical Bayes framework. This framework allows parameter values to vary between participants but also pulls parameter values toward estimates at the group level (see Appendix H for more details and estimated parameter values). Thus, hierarchical Bayes allows the strengths of the context effects to vary between participants, which has been previously reported (Berkowitsch, Scheibehenne, & Rieskamp, 2014; Tsetsos et al., 2017).

**Figure 9 fig9:**
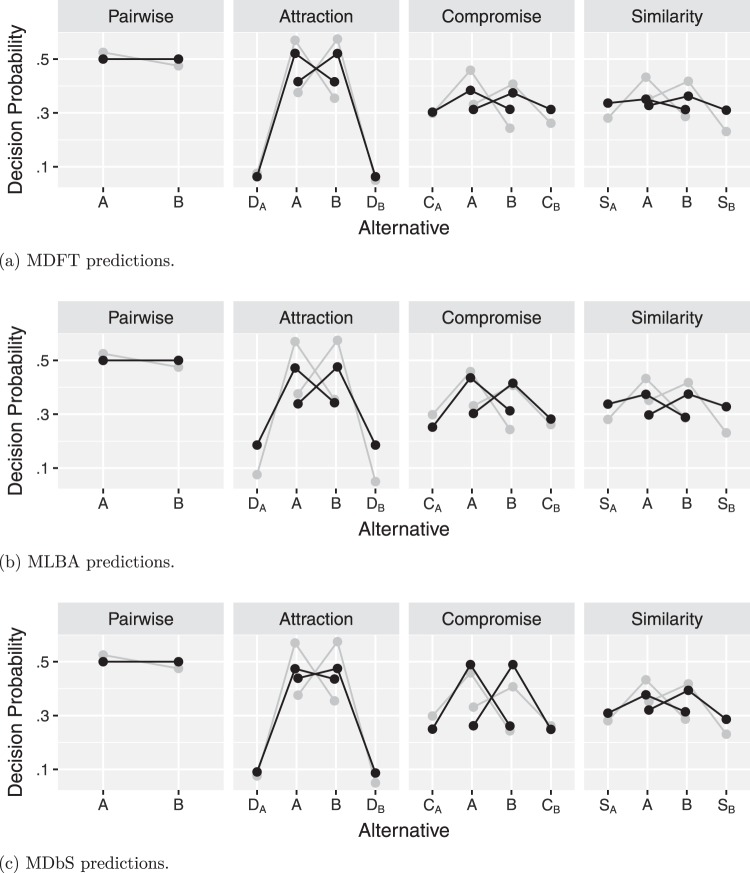
Mean predictions for the big-three consumer choices experiment, with the parameter estimates at the group level. Gray represents the data as shown in Figure 8.

With the parameter values estimated at the group level, we made predictions on the data with the three models. The results are summarized in Figure 9, which shows that the three models produce the qualitative patterns of context effects. Compared with the observed proportion of decisions (gray dots replicated from Figure 8), the MDFT model predicts strength of attraction effect quite well but tends to underestimate the similarity and compromise effects. The MLBA model, in contrast, predicts the compromise effect well but underestimates the attraction effect and, to a lesser extent, the similarity effect. Finally, the MDbS model predicts the compromise effect well but underestimates the attraction effect and, to a lesser extent, the similarity effect. Overall, however, none of the models appears to provide superior predictions across the three effects.[Fig-anchor fig9]

**Figure 10 fig10:**
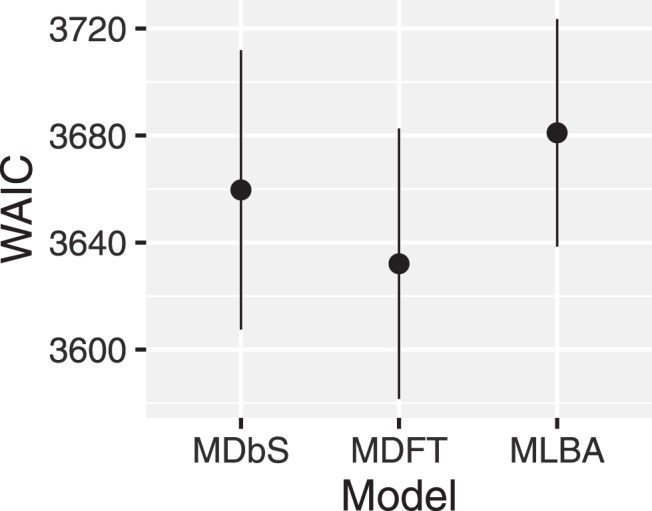
Model performance measured with the widely applicable information criteria (WAIC). Smaller value indicates better performance, and error bar represents 95% confidence interval. Overall, model performance is quite similar between the three models.

The performance of each model was quantitatively assessed with the widely applicable information criteria (WAIC; Watanabe, 2013; see also, Gelman, Hwang, & Vehtari, 2014). By using WAIC, we assess out-of-sample predictive accuracy: a model is favored if the model makes a better prediction for a new data point. An alternative approach, which we did not take, is to assess in-sample error: a model is favored if the model provides a better fit to the data we collected. This alternative approach often relies on BIC or Bayes factor (please see Gelman et al., 2013, for more discussion on the two approaches). WAIC is an estimate of expected predictive accuracy, and smaller values indicate that a model’s prediction for a new observation is likely to be more accurate. Thus, WAIC is larger for a model which over- or underfits the data. The results are summarized in Figure 10. Figure 10 shows overlapping error bars, indicating that in terms of performance, the MDFT, MLBA, and MDbS models are not distinguishable. One advantage for MDbS model is that it does not require attribute values to be on the same scale and unit, but can still achieve performance comparable with the MLBA and MDFT models.[Fig-anchor fig10]

Thus far, we have seen that the MDbS model can provide an account of the big three context effects. The mechanisms in the MDbS model were constrained by eye movement process data, but MDbS generalized well to choice proportions for the big-three choice phenomena. In fact, despite—or perhaps because of—these constraints, MDbS’s quantitative account is about the same as that offered by other the prominent accounts from the MDFT and MLBA models. Below we turn to the additional multialternative decision phenomena in the literature and consider the breadth of accounts offered by the MDbS, MDFT, MLBA, and componential context models.

### A Qualitative Account of the Breadth of Multialternative Decision Phenomena

In this section, we compare the models in their capabilities to explain a broad range of multialternative decision phenomena beyond the big three context effects. To identify other key phenomena, we surveyed theoretical studies which discuss at least two of big three context effects. All of the phenomena discussed in these studies are listed in Table 3. Thus Table 3 represents the range of phenomena of concern in the literature, and not a hand-picked list of phenomena that the MDbS model can explain. The first three rows concern experiments run by Stewart and colleagues which we have described above. The remaining rows are about experiments run by other researchers. We note in the main text and the footnotes to Table 4 where minor modifications might be made to theories to capture effects—otherwise, effects are captured by the “vanilla” models as presented here without any modification.[Table-anchor tbl3][Table-anchor tbl4]

With these choice sets, we examined whether a model explains the context effects by testing all of the possible combinations of reasonable parameter values (see Appendix J for more details). Then, we examined the maximum number of context effects a model can explain. Given the purpose of the existing models, we restrict our attention to the parameter values which produce the attraction and compromise effects for the componential context model (discussed below) and the attraction, compromise and similarity effects for the MDFT and the MLBA model.

As with the quantitative comparison above, we normalized the attribute values for all models, except the MDbS model which does not require this. The normalized attribute values are listed in Table J1 in Appendix J.

Below we describe the modeling of each phenomenon in detail. We have reused the single set of MDbS parameter values from earlier: α = 3.0, β_0_ = 0.1, β_1_ = 50, and θ = 0.1. Overall, the results highlight that the MDbS model predicts a wider range of context effects than the existing models. First though, we introduce the componential context model and briefly review other models.

#### The componential context model

We have included the componential context model (CCM; Tversky & Simonson, 1993) in the qualitative evaluation. We omitted it from the quantitative evaluation of the big three effects because the model does not account for the similarity effect (Roe et al., 2001) and because the model does not produce decision probabilities. The CCM was developed to explain the background contrast and the compromise effects. In the CCM, the subjective value of an alternative is an average of two quantities: a weighted sum of attribute values, which explains the background contrast effect; and a relative advantage of attribute values, which explains the attraction and compromise effects. The CCM produces subjective values for each alternative, and the alternative with the highest subjective value is chosen. Thus, unlike the other models we have discussed, the CCM does not implement an evidence accumulation process. As a result, the CCM does not explain the effects associated with time pressure. Previously, Soltani et al. (2012) simplified the CCM and show that the CCM predicts a stronger attraction effect with a closer decoy, but without the simplification, the CCM correctly predicts a weaker attraction effect with a closer decoy. The computational implementation of the CCM is described in Appendix I.

#### Other models

Other evidence accumulation models often require simulations to produce predictions. One simulation run of such model produces one decision. It takes of the order of 1,000 or more simulation runs to estimate decision probabilities with sufficient precision. Such models include the leaky competing accumulator model (Usher & McClelland, 2004) and the associative accumulation model (Bhatia, 2013). Other models, which this article does not review, include 2N-ary choice tree model (Wollschläger & Diederich, 2012) and range-normalization model (Soltani et al., 2012).

### The Alignability Effect

In the alignability effect, attributes that are shared over alternatives have a greater impact on decisions and valuations than attributes that are unique to single alternatives (Markman & Medin, 1995; Slovic & MacPhillamy, 1974; Zhang & Markman, 2001). For example, consider a choice between two microwave popcorns. Popcorn A is described in terms of calorie content and kernel size, and Popcorn B is described in terms of calorie content and sweetness of taste. The common calorie content attribute has greater impact on decisions than the unique kernel size and sweetness attributes.

The alignability effect has been explained with the notion of ease of comparison. A comparison between alternatives along the common dimension is considered cognitively easier, and this ease of comparison is considered to lead to greater reliance on the common dimension (Slovic & MacPhillamy, 1974).

In the MDbS model, this ease of comparison is related to the difference between attribute values that are already in working memory because they are part of the problem and attribute values that must be sampled from long-term memory. In the above example, a comparison on calories is relatively likely, because calorie values are available in working memory for both alternatives. In contrast, when evaluating alternatives on noncommon dimensions, people must sample relevant values from long-term memory, but people do not appear to always do this sampling from long-term memory (e.g., Kassam, Morewedge, Gilbert, & Wilson, 2011). People’s working memory, for example, may be already fully loaded with attribute values sampled from other alternatives in the choice set. Without sampling from long-term memory, the noncommon attributes will not be used in comparisons and will not contribute to the accumulation rates.

Further, there will be individual differences in the sampling. When evaluating popcorn’s sweetness, for example, some people may sample the extreme sweetnesses of candies from long-term memory, whereas others may sample the more subtle sweetness in fruits. Thus for some people the popcorn’s sweetness will be evaluated favorably and for others it will be evaluated unfavorably. Consequently, when averaged across people, attribute values on noncommon dimensions will not appear to explain people’s valuation and decisions.

### The Attribute Balance Effect

**Figure 11 fig11:**
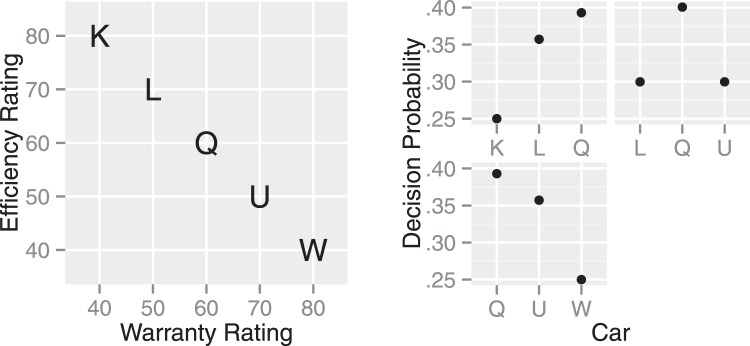
The attribute balance effect. Here, each car is rated on efficiency and warranty, both of which range from 0 to 100. Car Q is the balanced alternative with the same rating on both attributes (the left panel). The multialternative decision by sampling (MDbS) model predicts a higher decision probability for Car Q, when presented with Cars K and L, with Cars L and U, or with Cars U and W (the right panels).

The attribute balance effect is found when two attribute dimensions are on the same scale range and unit. An example is when available cars are rated on the scale from 0 to 100 for both warranty and fuel efficiency (see the left panel in Figure 11). Under this condition, people tend to decide on an alternative which has the same ratings for both attributes (Chernev, 2004, 2005).[Fig-anchor fig11]

This attribute balance effect has been attributed to people’s aversion to disperse values within an alternative (Chernev, 2005). For example, the attribute values for Car L in the left panel of Figure 11 differ from each other by 20 = 70 (efficiency rating) – 50 (warranty rating). This difference is considered to reduce the attractiveness of Car L. Thus, this account postulates that people collapse the attribute dimensions and compare attribute values across dimensions. In support, Chernev (2004) reports that when participants were primed to examine alternatives attribute by attribute and not to collapse the dimensions, their decisions do not show the attribute balance effect. In addition, when the attribute dimensions are not collapseable (e.g., because of different units), the attribute-balance effect is not observed (Chernev, 2004).

The MDbS model explains the attribute balance effect by allowing people to compare values across attribute dimensions when attribute dimensions are commensurable. The efficiency rating of Car L, for example, may be compared against the warranty rating of Car Q. When attribute dimensions are collapsed, the balanced alternative (i.e., Car Q) becomes the compromise alternative. In a choice set with Cars K, L, and Q in Figure 11, the attribute values after collapsing the dimensions are {40, 50, 60, 60, 70, 80}. The middle two values, 60, belong to Car L. Then, the attribute-balance effect emerges with the same mechanism as the compromise effect: Car Q is most frequently evaluated, leading to a higher decision probability for Car Q.

The explanation of the MDbS model is examined under the same conditions as the experiments reported by Chernev (2004) and Chernev (2005). Specifically, the attribute balance effect has been reported in choice sets with three alternatives: Cars K, L and Q; Cars L, Q, and U; and Cars Q, U, and W. Across the three choice sets, the MDbS model predicts the highest decision probability for the balanced alternative, Car Q (see the right panel in Figure 11).

The attribute balance effect is part of the motivation for the choice of subjective value function in the MLBA model. Thus the attribute balance effect is built into the MLBA model—there is no independent explanation of the effect.

### The Attribute Range Effect

In the attribute range effect (Mellers & Cooke, 1994), how attractive people find one attribute value depends on a range of values people previously saw in other choice sets. In one of the experiments, participants were asked to rate attractiveness of many apartments, each of which was described in terms of rent and commute time. In one condition, attribute values have a narrow range: for example, participants rated commute times ranging from 10 to 26 min. In another condition, attribute values have a wide range: participants rated commute times ranging from 1 to 50 min. The results show that a difference in attractiveness ratings between 10 min and 26 min was smaller when the commute time range was wider.

The attribute range effect is attributed to the people’s tendency to scale perceived attractiveness using the possible ranges in the values they saw (Mellers & Cooke, 1994). Suppose the perceived attractiveness ranges from 0 to 1, and that the commute time is linearly transformed onto this attractiveness scale. Then, when the commute time ranges from 1 to 50 min, the difference between 10 and 26 min commute covers about 30% ($=\frac{26-10}{50-1}$) of the range. In contrast when the commute time ranges from 10 to 26 min, the difference between 10 and 26 min commute covers 100%, the entire range. As a result, the difference in perceived attractiveness between 10 and 26 min commute is smaller when the commute time has a wider range.

This account is essentially identical to the relative rank account offered by the MDbS model. A relative rank can only range from 0 to 1, because it is the proportion of attribute values to which a target attribute is favorably compared. Thus, a difference in relative ranks between two fixed values tends to be smaller when attribute values in working memory have a wider range, because then fewer attribute attributes are positioned in between the two fixed values.

### The Attribute Spacing Effect

Similarly, in the attribute spacing effect (Cooke & Mellers, 1998) attractiveness ratings depend on spacing between values. For example, holding the range of commute times constant, people find a longer commute time like 18 min less attractive when attribute values are densely distributed between 10 and 13 min than when attribute values are densely distributed between 23 and 26 min.

This attribute spacing effect is attributed to range-frequency theory (Parducci, 1965). In range-frequency theory, attractiveness ratings depend on two factors: the relative position in the frequency distribution and the relative position in the range. The former, relative position in the frequency distribution, is a relative rank, and is sufficient to explain the attribute spacing effect. This account with relative ranks is identical to the MDbS’s explanation.

### Background Contrast Effects

**Figure 12 fig12:**
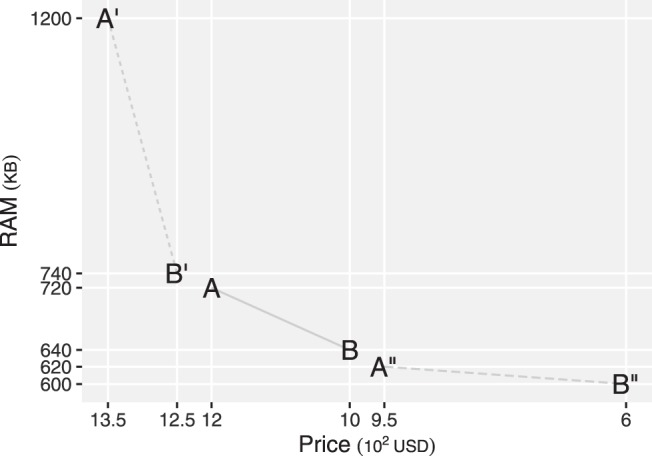
A set of alternative computers used in the experiment by Simonson and Tversky (1992). Participants first made a decision between A′ and B′ or between A″ and B″ and then were asked to make a decision between A and B.

Decisions are influenced by the trade-offs that people have made before. A set of alternatives used in an experiment reported in Simonson and Tversky (1992) is illustrated in Figure 12. The background contrast effect documents that a decision between A and B depends on whether people previously considered a decision between A′ and B′ or between A″ or B″.[Fig-anchor fig12]

This effect has been attributed to people’s tendency to learn a trade-off rate (Simonson & Tversky, 1992; Tversky & Simonson, 1993). A trade-off between A′ and B′ is at the rate of $0.22 per KB of RAM, while a trade-off between A and B is at the rate of $2.50 per KB of RAM. Thus after making a decision between A′ and B′, people may find the trade-off rate between A and B high and are less likely to seek additional RAM for additional price. As a result, people are less likely to choose a computer with larger RAM and higher price, A, than the other computer, B. In contrast, after making a decision between A″ and B″, where a trade-off is at the rate of $17.50 per KB of RAM, people may find the trade-off rate between A and B low and are likely to seek additional RAM for additional price. As a result, people are more likely to choose the computer with larger RAM and higher price, A, than the other computer, B.

The learning of trade-off rate is, however, not required for the MDbS model to explain this effect. When A′ and B′ are in working memory, the relative rank of A on RAM, where A is advantageous, decreases from 1.0 to .33, while relative ranks of B on price and RAM stay the same. As a result, the probability that Computer A is favored through comparison decreases, but the probability that Computer B is favored stays the same. Although B′ and A are similar to each other and hence, A is more frequently evaluated than B, the decrease in the probability of favorable evaluation more than offsets this and the MDbS model predicts a smaller decision probability for A (.38) than for B (.62; see Appendix C for how these decision probabilities are computed).

In contrast, when A″ and B″ are in working memory, relative ranks of A on price and RAM stay the same, while the relative rank of B on price, where B is advantageous, decreases from 1.0 to .33. As a result, the probability that Computer A is favored stays the same but the probability that Computer B is favored decreases. Again although A″ and B are similar to each other and hence, B is more frequently evaluated than A, the decrease in the probability of favorable evaluation more than offsets this and the MDbS model predicts a higher decision probability for A (.71) than for B (.29).

### The Centrality Effect

The centrality effect concerns the physical locations of alternatives where they are presented to people: when alternatives in a choice set are equally valuable, the alternative placed in the vertical or horizontal center is more likely selected (Christenfeld, 1995; Shaw, Bergen, Brown, & Gallagher, 2000). This centrality effect is linked to attention: people are more likely to attend an alternative located in the center (Atalay, Bodur, & Rasolofoarison, 2012; Shaw et al., 2000).

As the central alternative attracts more attention, the MDbS model predicts that the central alternative is more frequently evaluated. With an increasing frequency of evaluation, an alternative becomes more likely to accumulate evidence. This is because each alternative is equally valuable and hence, is equally likely to win a comparison. Thus, the MDbS model explains the centrality effect with the bias in frequency of evaluation.

### The Endowment Effect

The endowment effect concerns valuation of an object relative to valuation of another object people already own. In the famous mug experiment (Knetsch, 1989), participants were either given a mug or a chocolate bar, at random. Later they were given a costless and low effort opportunity to swap. No matter what the overall preference for a mug or a chocolate bar, half of people should be expected to swap—but few did. The classic explanation is that endowing someone with an object makes it intrinsically more valuable. This effect is also called the status quo bias (Tversky & Kahneman, 1991; see also, Samuelson & Zeckhauser, 1988).

The endowment effect has been explained with loss aversion. Compared with an object which people already own, a new object has better aspects and poorer aspects, and thus an exchange between the objects result in gain on some aspects and loss on the other aspects. Loss aversion means that the losses associated with the exchange will outweigh the gains (Kahneman, Knetsch, & Thaler, 1990). Further, when forced to make an exchange, people are more willing to forgo the object they own for a similar object than a dissimilar object to avoid a potentially large loss (Tversky & Kahneman, 1991).

In the MDbS model, ownership of an object is not expected to influence its evaluation. It could be that people sample different values from long-term memory, depending on what is in their possession. At the current formulation, however, the MDbS model does not provide an explanation for the endowment effect. We only note that the existence of the endowment effect is currently under dispute (e.g., Plott & Zeiler, 2005).

### The ‘Less Is More’ Effect

The ‘less is more’ effect can occur when an attribute, which people do not find particularly valuable, is added to one alternative in a choice set. This addition tends to reduce attractiveness for the alternative (Simonson, Carmon, & O’Curry, 1994). For example, the attractiveness of a car can be reduced after the car is bundled with a relatively unattractive branded umbrella. This effect has been attributed to an inference: people assume that the umbrella is only bundled with unattractive cars, and then that the car must be unattractive. This effect is also consistent with the information integration in impression formation, where people appear to take an average of attributes (Anderson, 1965, 1981).

In the MDbS model, however, the ‘less is more’ effect is explained within the comparison and accumulation processes. Introducing, or drawing attention to, an attribute that is not likely to win comparisons will reduce the accumulation rate for the alternative to which it belongs.

### The Perceptual Focus Effect

**Figure 13 fig13:**
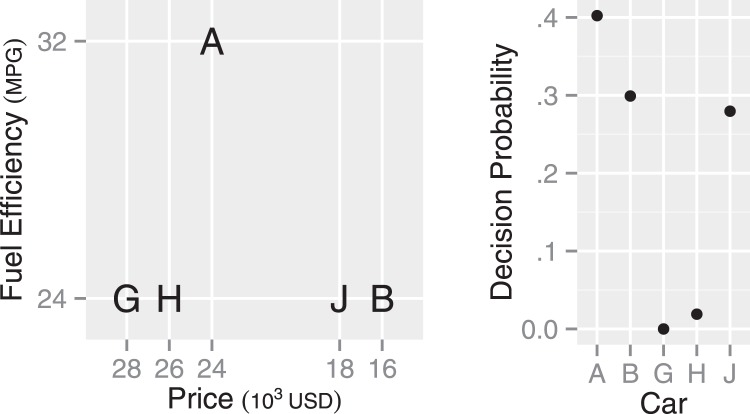
The perceptual focus effect. In line with empirical findings, the multialternative decision by sampling (MDbS) model predicts the highest decision probability for Car A.

The perceptual focus effect has been reported with the choice set illustrated in the left panel of Figure 13. Here, a previous study reports that Car A is most frequently chosen (Hamilton, Hong, & Chernev, 2007). In this choice set, importantly, Cars G, H, J and B share the same value on the fuel efficiency, making Car A distinctive on the fuel efficiency. This distinctiveness has been considered to facilitate people’s attention to be biased toward Car A, leading people to decide on Car A (Hamilton et al., 2007).[Fig-anchor fig13]

The biased attention is also predicted by the MDbS model. As the price of Car A is similar to two cars (Cars G and H), Car A is frequently compared on its price. About half of this frequent comparisons favors Car A, because price of Car A has a relative rank of .50. In contrast, the price of Car B has a higher relative rank of 1.0. Car B is, however, similar to only one car (Car J) and thus less frequently compared.

The biased attention on price, however, provides only an incomplete explanation by the MDbS model. On fuel efficiency, Car A has a relative rank of 1.00 and is always favored in the comparisons. In contrast, comparisons on fuel efficiency never favor the other cars. These differences in frequency of comparisons and relative ranks result in the highest decision probability for Car A (see the right panel in Figure 13).

### The Phantom Decoy Effect

**Figure 14 fig14:**
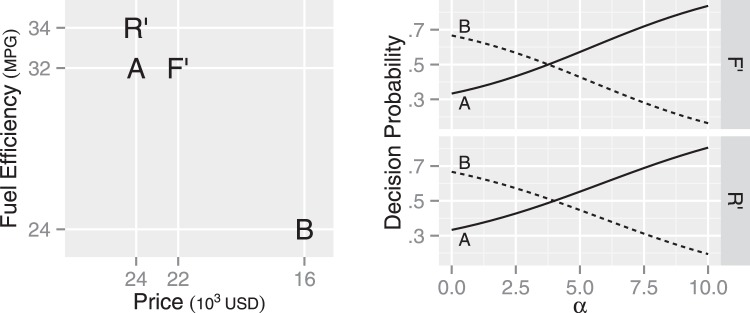
The phantom decoy effect. Here, a choice set contains either Cars A, B, and R′ or Cars A, B, and F′. Before people make a decision, Car R′ or F′ is announced unavailable. The right panels summarize predicted decision probabilities in the multialternative decision by sampling (MDbS) model, for the choice set with Cars A, B, and ″ (the top right panel) and the choice set with Cars A, B, and R′ (the bottom right panel).

The phantom decoy effect can occur when one alternative in a choice set is announced as unavailable. After this announcement, an alternative, which is similar but inferior to the now unavailable alternative, becomes more likely chosen than other alternatives (Highhouse, 1996). In a choice set with Cars A, B, and R’, for example, unavailability of Car R′ tends to make Car A more likely chosen than Car B (see Figure 14). Further, this effect is weaker in a choice set with Cars A, B, and F’, where Car F′ becomes an unavailable alternative (Pettibone & Wedell, 2007).[Fig-anchor fig14]

This phantom decoy effect has been explained with a combination of two factors (Pettibone & Wedell, 2007; Tsetsos et al., 2010): a change in reference point and loss aversion. After an alternative becomes unavailable, this alternative becomes a reference point against which other alternatives are compared. In the choice set with Cars A, B and R′, for example, Cars A and B are evaluated against Car R’. The disadvantage of some alternatives (i.e., Car B on fuel efficiency) become exaggerated due to loss aversion. As competitive but dissimilar alternatives involve offsetting a large loss against a large gain, loss aversion affects dissimilar alternatives more. The exaggerated disadvantage reduces the probability of selecting the alternatives with large losses (Car B in the above example).

The phantom decoy effect can potentially be explained with the MDbS model. Here, because the unavailable alternative is most similar to the alternative which is inferior to the unavailable alternative, the inferior alternative is most frequently evaluated. This more frequent evaluation leads to a higher accumulation rate and produce the phantom decoy effect. This explanation, however, depends on the extent to which the evaluation frequency is influenced by similarity between attribute values.

The phantom decoy effect as illustrated by the MDbS model is summarized in the right panels of Figure 14. In this illustration, we treat attribute values of the unavailable alternative as values in working memory. Here, parameter α dictates the influence of similarity on the evaluation frequency. When the parameter value is large and similarity has a strong influence on the evaluation frequency, the MDbS model shows the phantom decoy effect: the decision probability for Car A is higher than that for Car B. But note that we require the parameter α to be larger than for all of the other simulations in this paper. The MDbS model is unable to predict the weaker effect with Car F′ than with Car R′.

### The Polarization Effect

To illustrate the polarization effect, suppose that people are equally likely to decide on either of two cars: Car A with higher price and better efficiency, and Car B with lower price and poorer efficiency. In the polarization effect the addition of a compromise alternative, whose price and efficiency is between Cars A and B, to the choice set reduces the proportion of decisions made on Car B. The proportion of decisions made on Car A, however, is not affected. Consequently, Car A is most likely selected (Simonson & Tversky, 1992).

The polarization effect has been attributed to people’s selective extremeness aversion (Simonson & Tversky, 1992): people are averse to low efficiency but not to high price. As Simonson and Tversky (1992) point out, it is not clear why people show extremeness aversion in one attribute dimension but not in another. As a result, the explanation of this polarization effect requires additional parameterization, specifically tailored for this effect (Tversky & Simonson, 1993).

The MDbS model does not treat one attribute dimension differently to other dimensions, and thus, does not provide an explanation for this polarization effect. However, we note that this effect is not compatible with the compromise effect: in majority of the studies reported under the polarization effect (Simonson & Tversky, 1992), a compromise alternative is least frequently chosen, showing the opposite pattern to the compromise effect, where a compromise alternative is most frequently chosen. Thus, a model which can explain the compromise effect requires additional mechanisms to explain the polarization effect. Details of the additional mechanism await further research.

### Intransitive Preference Cycles

The MDbS model is readily applied to choices with more than two attribute dimensions, some of which may contain missing values. To illustrate, consider a choice between Alternatives V, Y, and Z in Table 5. The attribute values in Alternatives V, Y, and Z are systematically assigned, so that if a missing value is ignored, a pairwise comparison between Alternative V and Y favors Alternative Y, a pairwise comparison between Alternatives Y and Z favors Alternative Z, and a pairwise comparison between Alternative Z and V favors V. The MDbS model predicts a cycle of intransitive preference in the set of choices between two of the alternatives. Missing values are handled in the MDbS model with zero probability to evaluate an alternative on the attribute dimension where its value is missing. Alternative Z in Table 5, for example, has its price missing, so Alternative Z is never evaluated on its price. In a ternary choice, however, each alternative is equally likely to win a comparison, and the MDbS model predicts that no alternative is strongly preferred in a ternary choice. This intransitive pairwise choice but the indifferent ternary choice are reported by Müller-Trede, Sher, and McKenzie (2015).[Table-anchor tbl5]

## Related Models

Thus far, we have discussed how the MDbS model explains various phenomena with the four principles: (a) people sample relevant values from memory, (b) an alternative is evaluated through a series of pairwise ordinary comparisons, (c) the probability of comparing alternatives depends on similarity between attribute values, and (d) relatively small differences in attribute values are ignored. These principles of the MDbS model, however, are not entirely novel and have been implemented in existing models of decision making. In this section, we address how the MDbS model relates to other models.

Like MDbS, the exemplar-based random walk (EBRW) model (Nosofsky & Palmeri, 1997) implements the sampling from long-term memory. The EBRW model is a model of classification, where a new object is classified by sampling instances of contending categories from long-term memory. More similar instances are more likely to be retrieved more quickly, and each retrieved instance contributes one unit of evidence for its category. In MDbS, similarity also influences the comparison process and one unit of evidence is also accumulated at each time. In MDbS, however, comparisons are made on single dimension, and values across dimensions are not aggregated as they are in the EBRW model.

Comparisons are considered to be an integral part of decision processes by Simonson, Bettman, Kramer, and Payne (2013), who proposed that decisions are based on the comparisons which are task-acceptable and easy to make. According to Simonson et al. (2013), the task-acceptability depends on whether the comparison results are informative in judging which alternative is better. For example, when choosing between Cars A and B, a comparison between Cars A and X is not acceptable. This is because a comparison between Cars A and X does not justify a decision on Car A over B or Car B over A. Therefore, alternatives are compared only within a choice set. In the MDbS model in contrast, an alternative can be compared against attribute values sampled from long-term memory. The other component of Simonson et al.’s (2013) proposal, the ease of comparison, depends on a number of factors, including computational ease and saliency of alternatives. With this regard, we propose that similarity between attribute values also determines probability of evaluation.

In the MDbS model, the comparison of attribute values is insensitive to the magnitude of the differences, as long as the difference is judged meaningful. Magnitude-insensitive comparisons are implemented in some of the existing models. In fuzzy trace theory (Reyna, 2012), for example, a comparison is made on representations of attribute values. The fuel efficiency of 32 mpg, for example, can be represented as 32 mpg, *some* efficiency, or *better* efficiency. The latter two representations discretize the numerical values, and a comparison becomes magnitude-insensitive. This comparison with discrete representation also ignores small differences in attribute values: when 32 mpg and 29 mpg are both represented as *some* efficiency, the difference of 3 mpg disappears in the representation.

Similarly in a model proposed by de Clippel and Eliaz (2012), each alternative is ranked on each attribute dimension, and people decide on the alternative whose minimum rank is the highest. Thus, the procedure to rank alternatives is unbiased: unlike the MDbS model, probability to evaluate alternatives is not influenced by similarity between attribute values. de Clippel and Eliaz’s (2012) model, and MDbS, are closely related to the improper linear models of Dawes (1979), where regression weights are replaced with unit values of +1 or −1 and the tallying heuristic (Gigerenzer & Gaissmaier, 2011) in which favorable properties are just counted up.

The counting of favorable properties is also an integral part of query theory (Johnson, Häubl, & Keinan, 2007). In query theory, the decision making process proceeds by considering, in order, a number of queries and selecting the alternative favored by the most queries. The theory is applied, often, to experiments in which queries are rendered more or less accessible by experimental manipulations. In MDbS, the nature of the queries is different—they are binary ordinal comparisons typically between economic attribute values. MDbS also does not make strong assumptions about the ordering of the comparisons or queries, and has been applied to different kinds of phenomena in risky choice, intertemporal choice, and other multiattribute consumer choices.

The influence of similarity has also been implemented in models of risky decision (e.g., Buschena & Zilberman, 1999; Leland, 1994; Rubinstein, 1989). For example, Buschena and Atwood (2011) argue that people employ different decision strategies depending on the similarity between alternatives. Although in the MDbS model similar alternatives are evaluated in the same manner as dissimilar alternatives, the similarity between attribute values determines the probability of evaluation.

The use of a threshold below which differences are ignored is common in heuristic models. For example, among the models of risky decision, the priority heuristic (Brandstätter et al., 2006) implements the just meaningful difference. This heuristic predicts that people decide on the alternative if the alternative exceeds another by 10%. Brandstätter et al. (2006) argue that this 10% threshold is fixed. In contrast, the threshold is soft and probabilistic in the MDbS model. This probabilistic threshold has been implemented in models to explain how a change in prices influences consumer behavior (e.g., Han, Gupta, & Lehmann, 2001) and also to explain decisions on transportation (e.g., Cantillo, Heydecker, & Ortúzar, 2006; Cantillo & Ortúzar, 2006). And the logistic rule we use in Equation 4 for our soft threshold is a special case of the ubiquitous softmax function from probability theory, used in logistic regression and neural networks.

The principles in the MDbS model have been employed in various models. Our contribution to the exiting literature is to highlight that these principles are grounded in empirical findings, and to show that the combination of these principles explains the broad range of phenomena in preferential decisions.

## Conclusion

In this paper, we have extended the decision by sampling model to multialternative decisions. Our extensions are grounded in recent empirical findings from the process tracing literature. Specifically, we assume an evidence accumulation process where, in a series of comparisons, pairs of alternatives are compared on single dimensions, because empirical findings show that people’s eye movements comprise a series of alternations between pairs of attribute values. We also assume that more similar alternatives are selected for comparison more often, because empirical findings show that people attend to more similar alternatives more often. We assume that the rule for stopping evidence accumulation and making a decision is based on a relative comparison, because only a relative comparison is compatible with the gaze cascade effect and positively skewed response times. Despite, or perhaps because of, these process tracing constraints, the MDbS model provides a quantitative account of choice phenomena including the big three attraction, similarity, and compromise effects—an account equal to that of MDFT and the MLBA model. The MDbS model also provides the most comprehensive coverage of a survey of multialternative decision phenomena.

## Figures and Tables

**Table 1 tbl1:** Evidence Accumulation Models in Decision Making

Model	Evidence accumulated	Stochastic attention	Decision criterion
AAM	Transformed values on one attended attribute	One attribute is stochastically selected for each step of evidence accumulation	Absolute threshold
LCA	Differences in transformed attribute values, aggregated over attributes	Not assumed	External stopping time
MADDM	Pre-choice attractiveness ratings, weighted by visual attention	One alternative is selected for each step of evidence accumulation	Relative threshold
MDFT	Differences in attribute values between the alternative and the average of the other alternatives, on one attribute	One attribute is stochastically selected for each step of evidence accumulation	Relative threshold
MLBA	Differences in transformed attribute values, aggregated over attributes	Not assumed	Absolute threshold
RN	Transformed attribute values, aggregated over attributes	Not assumed	Not specified
MDbS	Ordinal comparisons between a pair of alternatives on single dimensions	A pair and an attribute are stochastically selected for each step of evidence accumulation	Relative threshold
*Note*. The model names are abbreviated as follows: AAM = associative accumulation model (Bhatia, 2013); LCA = leaky competing accumulators (Usher & McClelland, 2004); MADDM = multialternative attentional drift-diffusion model (Krajbich, Armel, & Rangel, 2010; Krajbich & Rangel, 2011); MDFT = multialternative decision field theory (Roe, Busemeyer, & Townsend, 2001); MLBA = multiattribute linear ballistic accumulator (Trueblood, Brown, & Heathcote, 2014); RN = range-normalization model (Soltani, De Martino, & Camerer, 2012); MDbS = multialternative decision by sampling.			

**Table 2 tbl2:** Comparisons Within the Choice Set Made in MDbS and Predicted Probability That a Comparison is Favorable to the Target

Choice set	Target	Comparison	Dimension	Probability of favorable comparison
Attraction	**A**	**B**	**Price**	**—**
			**Fuel efficiency**	**>.99**
	**A**	**D**	**Price**	**.78**
			**Fuel efficiency**	**.42**
	
	B	A	Price	>.99
			Fuel efficiency	—
	B	D	Price	>.99
			Fuel efficiency	—
	
	**D**	**A**	**Price**	**—**
			**Fuel efficiency**	**—**
	**D**	**B**	**Price**	**—**
			**Fuel efficiency**	**.98**

Compromise	**A**	**B**	**Price**	**—**
			**Fuel efficiency**	**>.99**
	**A**	**C**	**Price**	**>.99**
			**Fuel efficiency**	**—**
	
	**B**	**A**	**Price**	**>.99**
			**Fuel efficiency**	**—**
	B	C	Price	>.99
			Fuel efficiency	—
	
	C	A	Price	—
			Fuel efficiency	>.99
	C	B	Price	—
			Fuel efficiency	>.99

Similarity	A	B	Price	—
			Fuel efficiency	>.99
	A	S	Price	—
			Fuel efficiency	>.99
	**B**	**A**	**Price**	**>.99**
			**Fuel efficiency**	**—**
	
	**B**	**S**	**Price**	**.13**
			**Fuel efficiency**	**—**
	**S**	**A**	**Price**	**>.99**
			**Fuel efficiency**	**—**
	
	**S**	**B**	**Price**	**—**
			**Fuel efficiency**	**.05**
*Note*. Bold indicates comparisons which are more likely because the target is similar to the other alternatives. A dash (—) indicates a 0 probability of a favorable comparison.				

**Table 3 tbl3:** A List of Phenomena Collected From the Literature Review

Phenomenon	Tversky and Simonson (1993)	Pettibone and Wedell (2000)	Roe et al. (2001)	Usher and McClelland (2004)	Tsetsos, Usher, and Chater (2010)	Soltani et al. (2012)	Wollschläger and Diederich (2012)	Bhatia (2013)	Trueblood et al. (2014)	Tsetsos, Scheibehenne, Berkowitsch, Rieskamp, and Mata (2017)	Not discussed in previous work
Incidental value	—	—	—	—	—	—	—	—	—	—	✓
Attribute distribution	—	—	—	—	—	—	—	—	—	—	✓
Loss aversion	—	—	—	✓	—	—	✓	—	—	—	—
Attraction	✓	✓	✓	✓	✓	✓	✓	✓	✓	✓	—
Location of decoy	—	✓	—	—	—	—	—	✓	✓	—	—
Distance to decoy	—	✓	—	—	—	✓	—	—	—	—	—
Time pressure	—	—	—	—	—	—	—	✓	✓	—	—
Familiarity	—	—	—	—	—	—	—	—	—	—	✓
Correlation with the compromise effect	—	—	—	—	—	—	—	—	—	✓	—
Anti-correlation with the similarity effect	—	—	—	—	—	—	—	—	—	✓	—
Compromise	✓	✓	✓	✓	✓	✓	✓	✓	✓	✓	—
Time pressure	—	—	—	—	—	—	—	✓	✓	✓	—
Familiarity	—	—	—	—	—	—	—	—	—	—	✓
Anti-correlation with the similarity effect	—	—	—	—	—	—	—	—	—	✓	—
Similarity	—	—	✓	✓	✓	✓	✓	✓	✓	✓	—
Time pressure	—	—	—	—	—	—	—	—	✓	—	
Alignability	—	—	—	—	—	—	—	✓	—	—	—
Attribute balance	—	—	—	—	—	—	—	—	✓	—	—
Attribute range	—	✓	—	—	—	—	—	—	—	—	—
Attribute spacing	—	✓	—	—	—	—	—	—	—	—	—
Background contrast	✓	—	—	—	—	—	✓	—	—	—	—
Centrality	—	—	—	—	—	—	—	—	—	—	✓
Less is more	—	—	—	—	—	—	—	✓	—	—	—
Endowment	—	—	—	—	—	—	—	✓	—	—	—
Perceptual focus	—	—	—	—	—	—	—	—	—	—	✓
Phantom decoy	—	✓	—	—	✓	—	—	✓	—	—	—
Polarization	✓	—	—	—	—	—	—	—	—	—	—
*Note*. We have included three phenomena addressed in earlier articles on DbS (top rows). For completeness we have also included the effect of familiarity on the attraction and compromise effects, the centrality effect, and the perceptual focus effect. A check mark (✓) indicates which phenomenon were discussed in which articles.											

**Table 4 tbl4:** A Model-by-Phenomenon Matrix Where Check Marks (✓) Indicate That the Model Offers an Account of the Phenomenon

Phenomenon	Model			
	CCM	MDFT	MLBA	MDbS
Incidental value	—	—	—	✓
Attribute distribution	—	—	—	✓
Loss aversion	—	—	—	✓
Attraction	✓	✓	✓	✓
Location of decoy	✓	✓	✓	✓
Distance to decoy	✓	✓	✓	✓
Time pressure	—	✓	✓	✓
Familiarity	—	—	—	✓
Correlation with the compromise effect	—	✓	✓	✓
Anti-correlation with the similarity effect	—	✓	✓	✓
Compromise	✓	✓	✓	✓
Time pressure	—	✓	✓	✓
Familiarity	—	—	—	✓
Anti-correlation with the similarity effect	—	✓	✓	✓
Similarity	—	✓	✓	✓
Time pressure	—	—	✓	✓
Alignability	—	—	—	✓
Attribute balance	—	—	—	✓
Attribute range	—	—	—	✓
Attribute spacing	—	—	—	✓
Background contrast	✓	—	—	✓
Centrality	—	✓^b^	—	✓
Endowment	—	—	—	—
Less is more	—	✓^b^	—	✓
Perceptual focus	—	✓	✓	✓
Phantom decoy	—	✓^b^	✓^c^	✓^d^
Polarization	✓^a^	—	—	—
Intransitive preference cycles	—	✓	✓	✓
*Note*. Abbreviations of the model names are: CCM for the componential context model, MDFT for decision field theory, MLBA for the multiattribute linear ballistic accumulator model, and MDbS the multialternative decision by sampling.				
^a^ The CCM needs a different function for one of the attribute dimensions to produce the polarization effect. There is no a priori rule to select this dimension. ^b^ MDFT can be extended to explain these context effects (Tsetsos et al., 2010). ^c^ The MLBA model can produce the phantom decoy effect with additional parameterization (Trueblood et al., 2014). ^d^ The similarity parameter in MDbS needs to be larger to produce the phantom decoy effect.				

**Table 5 tbl5:** Alternatives V, Y, and Z to Illustrate the Cycle of Intransitive Preference

Measure	V	Y	Z
Price (10^3^ USD)	24	16	—
Fuel efficiency (MPG)	—	24	32
Warranty rating	70	—	50
*Note*. Dash (—) indicates a missing value.			

**Table F1 tbl6:** Attribute Values Used in the Experiment

Product	Dimension	Alternative	
		A	B
Mouthwash	Breath	4.5 hours	7.2 hours
	Germs killed	77%	56%
Exercise class	Fee	$9.49	$6.49
	Calories	356kcal	259kcal
Box of chocolate	Amount	26oz	33oz
	Variety	9	5
GPS	Update	3.04Hz	5.62Hz
	Accuracy	4.97m	7.83m
Mobile battery	Price	$19.93	$13.49
	Talk time	14.55 hours	9.25 hours
Light bulb	Life	1309 hours	1923 hours
	Price	$1.35	$2.50
Air purifier	Noise	64.7dB	39.3dB
	Efficiency	325cfm	203cfm
Strawberry	Quantity	407g	452g
	Price	$2.58	$2.85
*Note*. For the description of dimensions, please see the main text.			

**Table H1 tbl7:** Posterior Estimates of Mean Parameter Values at the Population Level

Model	Parameter	Median	95% HDI
MDFT	ϕ_1_	.03	.01, 2.99
	ϕ_2_	.12	−.04, .35
	σ	1.03	.58, 2.50
	ξ	20.80	3.87, 49.33
	T	36.72	22.76, 114.48
MLBA	m	41.27	−8.36, 72.08
	λ_1_	.68	.42, 22.63
	λ_2_	1.21	−5.31, 2.14
	*I*_0_	2.10	−.18, 40.08
MDbS	α	1.94	.15, 2.26
	β_0_	.40	.05, .42
	β_1_	53.73	2.93, 147.53
	θ	.52	.22, 1.32
*Note*. HDI stands for highest density interval.			

**Table J1 tbl8:** Attribute Values Used for the Qualitative Comparison

Alternative	Attribute value	
	Dimension *x*	Dimension *y*
A	2.00	3.00
B	3.00	2.00
D	1.75	2.75
D′	1.90	2.90
F	1.75	3.00
R	2.00	2.75
C	1.00	4.00
S	2.90	2.10
J	2.90	2.00
H	1.90	2.00
G	1.80	2.00
K	.50	4.50
L	1.50	3.50
Q	2.50	2.50
U	3.50	1.50
W	4.50	.50

**Figure E1 fig15:**
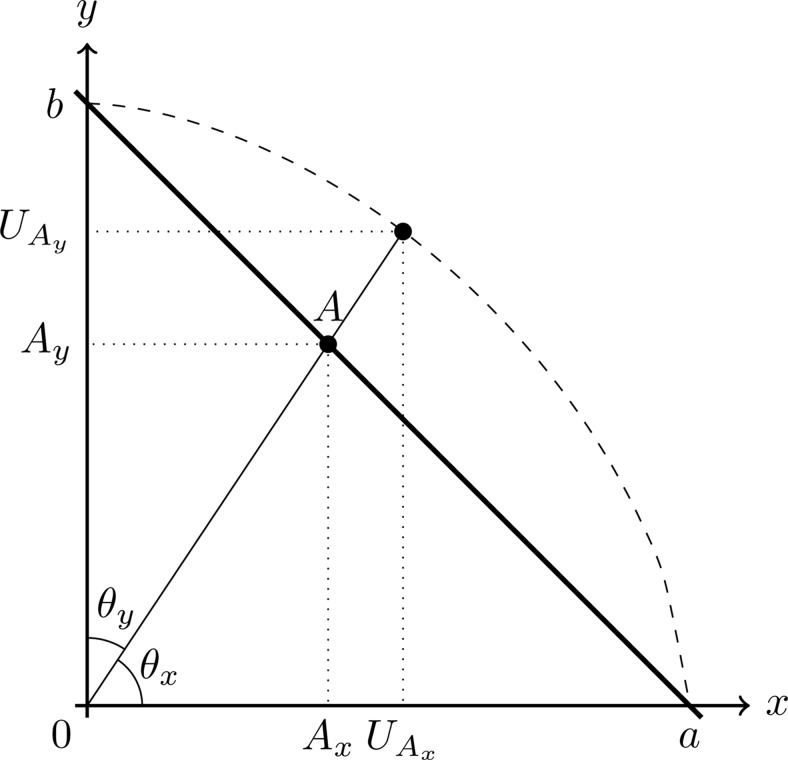
Illustration of the subjective value function in the MLBA. A bold, solid diagonal line represents indifference. The dashed arc represents ${\left(\frac{U_{A_{x}}}{a}\right)}^{m}+{\left(\frac{U_{A_{y}}}{b}\right)}^{m}=1$.

**Figure G1 fig16:**
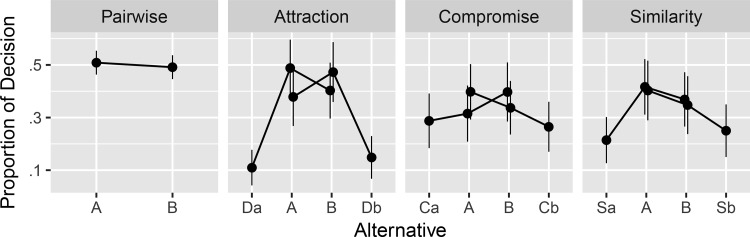
Choice proportions for participants excluded from the main analysis in the big-three consumer choices experiment. Each panel represents an experimental condition, a solid line connects a choice set, and error bars are 95% confidence intervals.

## A Random Walk Details

This Appendix describes a closed form solution for decision probabilities for MDbS. Over time, accumulators for each choice alternative are incremented by a series of pairwise, ordinal comparisons. The decision criterion is the difference between the maximum accumulator and the mean of all accumulators. Here we follow Diederich and Busemeyer (2003) and conceptualize the accumulation as a random walk over possible accumulator states.

For illustration purposes, suppose a choice set includes three alternatives, A, B, and D, and that one comparison is made and Alternative A is favored. Then, the accumulated evidence is 1 for Alternative A, and 0 for Alternatives B and D. The mean of this accumulated evidence is therefore 1/3. Thus, the relative evidence is 2/3 for Alternative A, and −1/3 for Alternatives B and D. The relative evidence is updated each time a pairwise ordinal comparison is made. When this relative evidence reaches the threshold θ, a decision is made.

For notation convenience, we multiply the relative evidences by the number of alternatives in a choice set, so that the relative evidences are always integers. We also multiply θ by the number of alternatives and round up to the nearest integer. In what follows, we use θ* to denote the transformed value of θ. When three alternatives are available, we define

$$\theta^{*}=ceil(3\theta ).$$ 5

By definition, the relative evidence always sums to 0. Leveraging this property, we can formulate the evidence accumulation with Markov chain, where a state is characterized by the relative evidence. A state can be, for example (2, −1, −1), where the relative evidence is 2 for Alternative A, −1 for Alternative B, and −1 for Alternative D.

The Markov chain is defined by the probability of transitioning from one state to another. This probability is organized in the following matrix *P* with four submatrices.

$$P=\left[\begin{array}{cc} I & 0\\ R & Q \end{array}\right],$$

where *R* is the transition matrix for reaching the decision criterion:

$$R=\begin{array}{cccccccc} \text{state} & & \left(\theta^{*},\theta^{*}-2,-2\theta^{*}+2\right) & \left(\theta^{*},\theta^{*}-3,-2\theta^{*}+3\right) & \cdots & \left(-2\theta^{*}+2,\theta^{*}-2,\theta^{*}\right) & \cdots & \\ \left(\theta^{*}-2,\theta^{*}-1,-2\theta^{*}+3\right) & & p_{A} & 0 & \cdots & 0 & \cdots & \\ \left(\theta^{*}-2,\theta^{*}-2,-2\theta^{*}+4\right) & & 0 & p_{A} & \cdots & 0 & \cdots & \\ \left(\theta^{*}-2,\theta^{*}-3,-2\theta^{*}+5\right) & [ & 0 & 0 & \cdots & 0 & \cdots & ]\\ \vdots & & & & & & & \\ \left(-2\theta^{*}+3,\theta^{*}-1,\theta^{*}-2\right) & & 0 & 0 & \cdots & p_{D} & \cdots & \\ \left(-2\theta^{*}+4,\theta^{*}-1,\theta^{*}-2\right) & & 0 & 0 & \cdots & 0 & \cdots & \end{array}$$

and *Q* is the transition matrix for evidence accumulation:

$$Q=\begin{array}{ccccccccccc} \text{state} & & . . . & \left(1,-2,1\right) & \left(-2,1,1\right) & \left(-2,-2,4\right) & \left(2,-1,-1\right) & \left(-1,2,-1\right) & \left(-1,-1,2\right) & \left(0,0,0\right) & \\ \vdots & & & & & & & & & & \\ \left(1,-2,1\right) & & \cdots & p_{0} & 0 & 0 & 0 & 0 & 0 & p_{B} & \\ \left(-2,1,1\right) & & \cdots & 0 & p_{0} & 0 & 0 & 0 & 0 & p_{A} & \\ \left(-2,-2,4\right) & & \cdots & 0 & 0 & p_{0} & 0 & 0 & 0 & 0 & \\ \left(2,-1,-1\right) & [ & \cdots & 0 & 0 & 0 & p_{0} & 0 & 0 & 0 & ]\\ \left(-1,2,-1\right) & & \cdots & 0 & 0 & 0 & 0 & p_{0} & 0 & 0 & \\ \left(-1,-1,2\right) & & \cdots & p_{A} & p_{B} & p_{D} & 0 & 0 & p_{0} & 0 & \\ \left(0,0,0\right) & & \cdots & 0 & 0 & 0 & p_{A} & p_{B} & p_{D} & p_{0} & \end{array}$$

Here, *p*_*A*_, *p*_*B*_ and *p*_*D*_ are the probabilities to accumulate evidence for Alternatives A, B, and D respectively, and *p*_0_ is the probability that none of the alternatives accumulate evidence. In the above matrices, a value on row *i* column *j* is the probability of moving from State *i* to State *j*.

To compute decision probabilities, we also need to define a vector, *Z*, to specify the starting state:

$$\begin{array}{l} \;\;\;\;\;\;\;\;\;\begin{array}{cccccccc} \cdots & \left(1,-2,1\right) & \left(-2,1,1\right) & \left(-2,-2,4\right) & \left(2,-1,-1\right) & \left(-1,2,-1\right) & \left(-1,-1,2\right) & \left(0,0,0\right) \end{array}\\ Z=\left[\begin{array}{cccccccc} \cdots & 0 & 0 & 0 & 0 & 0 & 0 & 1 \end{array}\right]. \end{array}$$

Then, the stationary probability of each state is given by

$$Z{\left(I-Q\right)}^{-1}R.$$

The decision probability is derived by summing the relevant cells in this stationary probability vector. For a more detailed description of this approach, please see the tutorial by Diederich & Busemeyer (2003).

## B Example Computation for the Attraction Effect

In this section, we describe the computation involved in the MDbS account of the attraction effect: The choice set contains Cars A, B, and D in Figure 3. We use subscripts _*x*_ and _*y*_ to denote the price and the fuel efficiency, and we ensure that a larger value indicates a better value by negating the price, such that *A_x_* = −24,000, *B_x_* = −16,000, *D_x_* = −27,000, *A_y_* = 32, *B*_*y*_ = 24, and *D*_*y*_ = 29.

First, we compute distances between the attribute values:

$$D_{\left(A_{i},X_{i}\right)}=\frac{\left|A_{i}-X_{i}\right|}{\left|X_{i}\right|}.$$

The computed distances are organized in matrices, such that the cell at Column *A*_*x*_ and Row *B*_*x*_ represents the distance from *A*_*x*_ to *B*_*x*_ (i.e., $D_{\left(A_{x},B_{x}\right)}$):

$$\begin{array}{l} D=\begin{array}{ccccccccc} & & A_{x} & B_{x} & D_{x} & A_{y} & B_{y} & D_{y} & \\ A_{x} & & - & \frac{\left|A_{x}-B_{x}\right|}{\left|B_{x}\right|} & \frac{\left|A_{x}-D_{x}\right|}{\left|D_{x}\right|} & - & - & - & \\ B_{x} & & \frac{\left|B_{x}-A_{x}\right|}{\left|A_{x}\right|} & - & \frac{\left|B_{x}-D_{x}\right|}{\left|D_{x}\right|} & - & - & - & \\ D_{x} & [ & \frac{\left|D_{x}-A_{x}\right|}{\left|A_{x}\right|} & \frac{\left|D_{x}-B_{x}\right|}{\left|B_{x}\right|} & - & - & - & - & ]\\ A_{y} & & - & - & - & - & \frac{\left|A_{y}-B_{y}\right|}{\left|B_{y}\right|} & \frac{\left|A_{y}-D_{y}\right|}{\left|D_{y}\right|} & \\ B_{y} & & - & - & - & \frac{\left|B_{y}-A_{y}\right|}{\left|A_{y}\right|} & - & \frac{\left|B_{y}-D_{y}\right|}{\left|D_{y}\right|} & \\ D_{y} & & - & - & - & \frac{\left|D_{y}-A_{y}\right|}{\left|A_{y}\right|} & \frac{\left|D_{y}-B_{y}\right|}{\left|B_{y}\right|} & - & \end{array}\\ =\begin{array}{ccccccccc} & & A_{x} & B_{x} & D_{x} & A_{y} & B_{y} & D_{y} & \\ A_{x} & & - & 0.50 & 0.11 & - & - & - & \\ B_{x} & & 0.33 & - & 0.41 & - & - & - & \\ D_{x} & [ & 0.13 & 0.69 & - & - & - & - & ]\\ A_{y} & & - & - & - & - & 0.33 & 0.10 & \\ B_{y} & & - & - & & 0.25 & - & 0.17 & \\ D_{y} & & - & - & & 0.09 & 0.21 & - & \end{array} \end{array}.$$

Please note that a comparison is not made against the same value (e.g., *A*_*x*_ against *A*_*x*_), so that we do not compute self-distance (e.g., $D_{\left(A_{x},A_{x}\right)}$).

Using the distances calculated above, we compute similarity between the attribute values:

$$S_{\left(A_{i},X_{i}\right)}=\exp\left(-\alpha \;D_{\left(A_{i},X_{i}\right)}\right).$$

In this example, we use α = 3. Thus,

$$S=\begin{array}{ccccccccc} & & A_{x} & B_{x} & D_{x} & A_{y} & B_{y} & D_{y} & \\ A_{x} & & - & 0.22 & 0.72 & - & - & - & \\ B_{x} & & 0.37 & - & 0.29 & - & - & - & \\ D_{x} & [ & 0.68 & 0.13 & - & - & - & - & ]\\ A_{y} & & - & - & - & - & 0.37 & 0.74 & \\ B_{y} & & - & - & - & 0.47 & - & 0.60 & \\ D_{y} & & - & - & - & 0.76 & 0.53 & - & \end{array}.$$

The row-wise sum of this matrix represents un-normalized probability to evaluate each attribute value. After dividing with the total sum of 5.80, we get *p*(evaluate *A_x_*) = 0.16, *p*(evaluate *B_x_*) = 0.11, *p*(evaluate *D_x_*) = 0.14, *p*(evaluate *A_y_*) = 0.19, *p*(evaluate *B_y_*) = 0.18, and *p*(evaluate *D_y_*) = 0.22.

We now compute the probability that each attribute wins a comparison, which is given by

$$p(A_{i}\text{ is favored over}\;X_{i})=\{\begin{array}{ll} F\left(\beta_{1}\;\left(D_{\left(A_{i},X_{i}\right)}-\beta_{0}\right)\right) & \;\text{ if }A_{i}>X_{i}\\ 0 & \;\text{ otherwise}, \end{array}$$

where F is a sigmoid function. We use the logistic function, $F(z)=\frac{1}{1+\exp(-z)}$, and we set β_0_ = 0.1 and β_1_ = 50. The probabilities are organized in a matrix:

$$G=\begin{array}{ccccccccc} & & A_{x} & B_{x} & D_{x} & A_{y} & B_{y} & D_{y} & \\ A_{x} & & - & 0.00 & 0.64 & - & - & - & \\ B_{x} & & 1.00 & - & 1.00 & - & - & - & \\ D_{x} & [ & 0.00 & 0.00 & - & - & - & - & ]\\ A_{y} & & - & - & - & - & 1.00 & 0.54 & \\ B_{y} & & - & - & - & 0.00 & - & 0.00 & \\ D_{y} & & - & - & - & 0.00 & 1.00 & - & \end{array}.$$

Then, the probability that each value wins a comparison is computed as

$$p(A_{i}\text{ wins a comparison})=\underset{X_{i}\in {𝕊}_{i}}{\sum }p(A_{i}\text{ is compared against}\;X_{i})\;p(A_{i}\text{ is favored over}\;X_{i}).$$

This is achieved by taking the row-wise mean of the G matrix above:

$$\begin{array}{c} p(A_{x}\;wins\;a\;comparison)=0.32\;,\;\;p(B_{x}\;wins\;a\;comparison)=1.00,\\ p(D_{x}\;wins\;a\;comparison)=0.00,\;\;\;p(A_{y}\;wins\;a\;comparison)=0.77,\\ p(B_{y}\;wins\;a\;comparison)=0.00,\;\;p(D_{y}\;wins\;a\;comparison)=0.50. \end{array}$$

Finally, the accumulation rate is given by

$$p(\text{Evidence is accumulated toward }A)=\underset{i\in 𝔻}{\sum }p(\text{evaluate}\;A_{i})\;p(A_{i}\text{ wins a comparison}).$$

Thus, we obtain *p*(Evidence is accumulated toward *A*) = 0.20, *p*(Evidence is accumulated toward *B*) = 0.11, and *p*(Evidence is accumulated toward *D*) = 0.11.

When one unit of evidence is accumulated toward an alternative, the relative evidence—the accumulated evidence minus the mean evidence—is $1-\frac{1}{\text{ the number of alternatives}}$. When this value is greater than threshold θ, a decision criterion is satisfied soon as one unit of evidence is accumulated toward one alternative. In such case, decision probability is given by normalizing the accumulation rate. Otherwise, the decision probability is computed as described in Appendix A.

With θ = 0.10, one unit of evidence is sufficient for a decision criterion to be satisfied. Thus, the decision probability is .47 for Car A, .27 for Car B, and .26 for Car D.

## C Example Computation for the Background Contrast Effect

In this section, we describe the computation involved in MDbS when attribute values from previous choice sets are included in working memory. As an example, we take the background contrast effect: a choice is being made between Computers A and B, while the attribute values of Computers A′ and B′ are in working memory. Here, we use subscripts _*x*_ and _*y*_ to denote the price and RAM, so that *A_x_* = −1,200, *B_x_* = −1,000, *A*_*x*_′ = −1,350, *B*_*x*_′ = −1,250, *A*_*y*_ = 720, *B_y_* = 640, *A*_*y*_′ = 1,200, and *B*_*y*_′ = 740.

Then, the distances between the attribute values are

$$D=\;\;\begin{array}{ccccccccccc} & & A_{x} & B_{x} & A_{y} & B_{y} & {A^{\prime }}_{x} & {B\prime }_{x} & {A\prime }_{y} & {B\prime }_{y} & \\ A_{x} & & - & 0.2 & - & - & 0.11 & 0.04 & & & \\ B_{x} & [ & 0.17 & - & - & - & 0.26 & 0.20 & & & ]\\ A_{y} & & - & - & - & 0.12 & - & - & 0.40 & 0.03 & \\ B_{y} & & - & - & 0.11 & - & - & - & 0.47 & 0.14 & \end{array},$$

and the similarity is

$$S=\;\;\begin{array}{ccccccccccc} & & A_{x} & B_{x} & A_{y} & B_{y} & {A\prime }_{x} & {B\prime }_{x} & {A\prime }_{y} & {B\prime }_{y} & \\ A_{x} & & - & 0.55 & - & - & 0.72 & 0.89 & - & - & \\ B_{x} & [ & 0.61 & - & - & - & 0.26 & 0.55 & - & - & ]\\ A_{y} & & - & - & - & 0.69 & - & - & 0.30 & 0.92 & \\ B_{y} & & - & - & 0.72 & - & - & - & 0.25 & 0.67 & \end{array}.$$

After dividing the row-wise sum of S matrix with the total sum of 7.31, we get *p*(evaluate *A_x_*) = 0.29, *p*(evaluate *B_x_*) = 0.22, *p*(evaluate *A_y_*) = 0.26, and *p*(evaluate *B_y_*) = 0.22.

We now compute the probability that each attribute wins a comparison:

$$G=\;\;\begin{array}{ccccccccccc} & & A_{x} & B_{x} & A_{y} & B_{y} & {A\prime }_{x} & {B\prime }_{x} & {A\prime }_{y} & {B\prime }_{y} & \\ A_{x} & & - & 0.00 & - & - & 0.64 & 0.05 & - & - & \\ B_{x} & [ & 0.97 & - & - & - & 1.00 & 0.99 & - & - & ]\\ A_{y} & & - & - & - & 0.78 & - & - & 0.00 & 0.00 & \\ B_{y} & & - & - & 0.00 & - & - & - & 0.00 & 0.00 & \end{array}.$$

Then, the probability that each value wins a comparison is computed by taking the row-wise mean of the G matrix: *p*(*A_x_* wins a comparison) = 0.23, *p*(*B_x_* wins a comparison) = 0.99, *p*(*A_y_* wins a comparison) = 0.26, and *p*(*B_y_* wins a comparison) = 0.22.

Finally, we obtain *p*(Evidence is accumulated toward *A*) = 0.13 and *p*(Evidence is accumulated toward *B*) = 0.22. With θ = 0.1, a decision criterion is satisfied soon as one unit of evidence is accumulated toward one alternative. Then, the decision probability is .38 and .62 for Computers A and B, respectively.

## D The MDFT Model

In MDFT, the evidence for each alternative is accumulated over time. Here, we assume *N*_*a*_ alternatives, each of which is described in terms of *N*_*d*_ attribute dimensions, in a choice set. Then, accumulated evidence is organized in a *N*_*a*_ × 1 matrix, *P*, and evidence accumulation is formulated as follows:

P(t+1)=SP(t)+V(t+1),

where *S* is a *N*_*a*_ × *N*_*a*_ feedback matrix and *V* is a *N*_*a*_ × 1 momentary valence vector.

The feedback matrix, *S*, characterizes distance-dependent lateral inhibition between alternatives. The feedback from Alternative *A* to *B*, for example, is computed as:

$$-\phi_{2}\exp(\phi_{1}D_{AB}^{2}).$$

Here, *D*_*AB*_ is a distance between Alternatives *A* and *B*, which is defined as:

$$D_{AB}=\frac{{\left(A_{x}-A_{y}-B_{x}+B_{y}\right)}^{2}}{2}+\xi \frac{{\left(A_{x}+A_{y}-B_{x}-B_{y}\right)}^{2}}{2}.$$

The self feedback is computed as 1 − ϕ_2_.

The momentary valence vector is computed with four matrices:

V(t)=CMW(t)+Cϵ(t).

Here, *C* is a *N*_*a*_ × *N*_*a*_ matrix whose diagonal elements are 1 and off-diagonal elements are $\frac{1}{N_{a}-1}$, and *M* is a *N*_*a*_ × *N*_*d*_ matrix with attribute values, with each row corresponding to values of an alternative and each column corresponding to an attribute dimension. The attention weight *W* is a *N*_*d*_ × 1 vector, whose element is all 0 but one: when the first dimension is attended, the first element in *W* is 1 and all of the other elements are 0. We assume that all of the dimensions are equally likely attended. Finally, ϵ is a *N*_*a*_ × 1 vector with independent Gaussian noise whose variance is σ^2^.

For computational tractability, we assumed an external stopping rule to compute decision probability: a decision is assumed to be made after *T* steps of evidence accumulation. As decision probability depends on *T* (Busemeyer & Johnson, 2004; Roe et al., 2001), we treat *T* as a free parameter.

## E The MLBA Model

In the MLBA, evidence for each alternative is accumulated over time, and the rate of accumulation is determined by the drift rate. When Alternatives A, B, and D are in a choice set, for example, the drift rate for Alternative A is computed as follows:

$$d_{A}=V_{AB}+V_{AD}+I_{0},$$

where *V*_*AB*_ is the value of Alternative A relative to Alternative B. This relative value is given by the weighted sum of differences in the subjective values. The relative value of Alternative A over B on dimension *x*, for example, is given by

$$V_{AB}=W_{A_{x}B_{x}}\left(U_{A_{x}}-U_{B_{x}}\right)+W_{A_{y}B_{y}}\left(U_{A_{y}}-U_{B_{y}}\right),$$

where

$$W_{A_{x}B_{x}}=\{\begin{array}{ll} \exp\left(-\lambda_{1}|U_{A_{x}}-U_{B_{x}}|\right) & \text{if }U_{A_{x}}\geq U_{B_{x}},\\ \exp\left(-\lambda_{2}|U_{A_{x}}-U_{B_{x}}|\right) & \text{otherwise}. \end{array}$$

To compute the subjective values, we need to find the line of indifference on the attribute space (the solid diagonal line in Figure E1). Assuming that the attribute values are on the same unit, the line of indifference is determined by the sum of attribute values. If *A*_*x*_ + *A*_*y*_ is equal to *B*_*x*_ + *B*_*y*_. Alternatives A is considered indifferent from Alternative B. The line of indifference for Alternative A, for example, intersects the *x* axis at *a* = *A*_*x*_ + *A*_*y*_ and the *y* axis at *b* = *A*_*x*_ + *A*_*y*_.[Fig-anchor fig15]

The subjective value is the one that satisfies the following:

$${\left(\frac{U_{A_{x}}}{a}\right)}^{m}+{\left(\frac{U_{A_{y}}}{b}\right)}^{m}=1.$$

To find the subjective values, we need to compute the two angles:

$$\theta_{x}=\arctan\left(\frac{A_{y}}{A_{x}}\right)\text{ and }\theta_{y}=\arctan\left(\frac{A_{x}}{A_{y}}\right).$$

Then, the subjective values are given by

$$U_{A_{x}}=\frac{b}{{\left[\tan^{m}\left(\theta_{x}\right)+{\left(\frac{b}{a}\right)}^{m}\right]}^{1/m}}\text{ and}\;U_{A_{y}}=\frac{a}{{\left[\tan^{m}\left(\theta_{y}\right)+{\left(\frac{a}{b}\right)}^{m}\right]}^{1/m}.}$$

This subjective value function may seem different from what appeared in Trueblood et al. (2014), which described $U_{A_{y}}$ with θ_x_ and not with θ_y_. To see the equivalence, note that

$$\tan\left(\theta_{x}\right)=\tan\left(\arctan\left(\frac{A_{y}}{A_{x}}\right)\right)=\frac{A_{y}}{A_{x}}={\left[\frac{A_{x}}{A_{y}}\right]}^{-1}=\tan^{-1}\left(\arctan\left(\frac{A_{x}}{A_{y}}\right)\right)=\tan^{-1}\left(\theta_{y}\right).$$

Then,

$$U_{A_{y}}=\frac{a}{{\left[\tan^{m}\left(\theta_{y}\right)+{\left(\frac{a}{b}\right)}^{m}\right]}^{1/m}}=\frac{a}{{\left[\tan^{-m}\left(\theta_{x}\right)+{\left(\frac{b}{a}\right)}^{-m}\right]}^{1/m}}=\frac{a\tan\left(\theta_{x}\right)\frac{b}{a}}{{\left[\tan^{-m}\left(\theta_{x}\right)+{\left(\frac{b}{a}\right)}^{-m}\right]}^{1/m}\tan\left(\theta_{x}\right)\frac{b}{a}}=\frac{b\tan\left(\theta_{x}\right)}{{\left[{\left(\frac{b}{a}\right)}^{m}+\tan^{m}\left(\theta_{x}\right)\right]}^{1/m},}$$

which Trueblood et al. (2014) have in their Appendix C. In the implementation of the MLBA, $U_{A_{y}}$ is expressed with $U_{A_{x}}$:

$$U_{A_{y}}=\frac{b\tan\left(\theta_{x}\right)}{{\left[{\left(\frac{b}{a}\right)}^{m}+\tan^{m}\left(\theta_{x}\right)\right]}^{1/m}}=b{\left(\frac{\tan^{m}\left(\theta_{x}\right)}{{\left(\frac{b}{a}\right)}^{m}+\tan^{m}\left(\theta_{x}\right)}\right)}^{1/m}=b{\left(1-\frac{{\left(\frac{b}{a}\right)}^{m}}{{\left(\frac{b}{a}\right)}^{m}+\tan^{m}\left(\theta_{x}\right)}\right)}^{1/m}=b{\left(1-{\left(\frac{U_{A_{x}}}{a}\right)}^{m}\right)}^{1/m}.$$

The MLBA parameters are *m*, λ_1_, λ_2_, and *I*_0_.

## F Attribute Values Used in the Experiment

The attribute values used in the experiment are listed in Table F1. To explain each dimension, a short description was provided to participants with each choice. These descriptions are listed below.[Table-anchor tbl6]

### Mouthwash

Suppose you are about to buy a new mouthwash.

The attributes to consider are the number of hours that your breath stays fresh after rinsing and the percentage of germs that the mouthwash kills.

### Exercise Class

Suppose you are going to choose an exercise class.

The class fee and the average calories burned per class are described.

### Box of Chocolate

Imagine you are about to buy a box of chocolate.

The variety and the total amount (in ounces) are described.

### GPS

Imagine you are going to buy a GPS navigation system.

The position accuracy (in meters) and the update frequency (in Hz) are described.

The shorter the accuracy is, the GPS can more precisely locate where it is. Also, the higher the frequency is, the more easily the GPS can identify where it is moving.

### Mobile Battery

Imagine you are going to buy a replacement battery for your cell phone.

Below are the talk time battery life (in hours) and the price.

### Light Bulb

Imagine you are going to buy a new light bulb.

The usage life (in hours) and the price are as follows.

### Air Purifier

Suppose you are about to buy an air purifier.

Three purifiers are described in terms of the effectiveness (in cubic feet per minute) and the noise level (in decibels).

### Strawberry

Suppose you are going to buy a pack of strawberries at the supermarket.

The quantity (in grams) and the price are described below.

## G Additional Exploratory Analysis on the Choice Response Data

In this section, we report additional analysis on the choice response data requested by a reviewer. We did not plan to conduct this analysis in advance of data collection. Thus, the results reported in this section should be considered to be exploratory rather than confirmatory.

In particular, we explored whether our a priori exclusion criteria were retrospectively justifiable, by examining the difference in the choice responses between the participants whose data we included in the main analysis and the other participants. For this, we fit mixed-effect logistic regressions to predict a decision on Alternative A against Alternative B. The regression models have two independent variables: a binary variable to indicate the participant exclusion (i.e., whether a participant’s responses were included in the main analysis); and the context (whether the context favored Alternative A or B). The effect of the context is allowed to vary between attraction, compromise, and similarity choices. The likelihood ratio test indicates that the effect of the context differs between the participant groups: β = 0.75, χ^2^(1) = 9.00, *p* =.0028.

The data from the participants who were excluded from the main analysis are summarized in Figure G1. The figure shows that the attraction effect is observed: Alternative A is more often chosen when Alternative *D*_*A*_, which is inferior to A, was present then when Alternative *D*_*B*_, which is inferior to B, is present. The compromise and similarity effects are not clearly seen in Figure G1.[Fig-anchor fig16]

Thus, the exploratory analysis highlights the difference in choice proportions between participants who passed versus failed our attention check of avoiding a dominated alternative in our control choices.

## H Technical Details for Model Fitting

With hierarchical Bayes framework, we let parameter values vary between participants. In particular for parameters which can take only integers, we let values be Poisson distributed at participant level. The prior for this Poisson distribution (i.e., prior for group-level estimate) is set to non-informative distribution: *Gamma*(0, 0). For parameters which can take real values, we let values be normally distributed at participant level. The prior for this normal distribution (i.e., prior for group-level estimate) is also set to non-informative: *Normal*(0, ∞) for parameter mean, and *Uniform*(0, ∞) for standard deviation.

For each model, we used Markov chain Monte Carlo to draw 20,000 parameter values from the posterior distributions. Then we discarded the first 10,000 samples as the burn-in and thinned the remaining 10,000 samples to retain 1,000 samples. This procedure is repeated four times, leaving us with 4,000 samples for each model. The 4,000 samples for the group level estimates are summarized in Table H1.[Table-anchor tbl7]

## I The CCM Model

In the CCM, subjective value of an alternative is determined by the trade-off rate learned from previous decisions and the relative advantage within a choice set. When there is no previous decision to learn a trade-off, subjective value in the CCM reduces down to the sum of attribute values and the relative advantage. The subjective value for Alternative A, for example, is computed as follows:

$$\underset{i}{\sum }A_{i}+\theta \;\frac{Advantage(A)}{Advantage(A)+Disadvantage(A),}$$ 6

and

$$Advantage(A)=\underset{i}{\sum }\underset{X_{i}\in {𝕊}_{i}}{\sum }Advantage_{\left(i\right)}(A_{i},X_{i})$$ 7

where ${𝕊}_{i}$ is a set of attribute values on dimension *i* in a choice set. The advantage of A over X along dimension *i* is given by:

$$Advantage_{\left(i\right)}(A_{i},X_{i})=\{\begin{array}{ll} A_{i}-X_{i} & \;\text{ if }A_{i}>X_{i}\\ 0 & \;\text{ otherwise}. \end{array}$$ 8

Similarly, the disadvantage of A is computed as:

$$Disadvantage(A)=\delta \left(\underset{i}{\sum }\underset{X_{i}\in {𝕊}_{i}}{\sum }Advantage_{\left(i\right)}(X_{i},A_{i})\right).$$ 9

The disadvantage function δ is an increasing convex function, which satisfies δ(*t*) > *t*. Here, we use a convex function (i.e., δ(*t*) = λ log(*t*)). Previously, Soltani et al. (2012) used a linear function for δ: namely δ(*t*) = λ*t*, (λ > 1) and report that the CCM predicts a stronger attraction effect with a closer decoy. With δ(*t*) = λlog(*t*), however, the CCM predicts a weaker attraction effect with a closer decoy. We also note that when δ(*t*) = λ *t*, the CCM does not produce the compromise effect, which the CCM is designed to explain.

## J Additional Details for the Qualitative Comparison

The attribute values used in the qualitative comparison are listed in Table J1. The attraction effect is assessed with Alternatives A, B, and D, and is considered to be present when decision probability for Alternative A is higher than the others. Similarly, the compromise effect is assessed with Alternatives A, B, and C, the similarity effect is assessed with Alternatives A, B, and S, the perceptual distinctiveness effect is assessed with Alternatives A, B, J, H, and G, and the attribute balance effect is assessed with Alternatives K, L, Q, U, and W.[Table-anchor tbl8]

Some of the phenomena we discuss are not quantitatively specified well enough to allow us to simulate. These phenomena are the alignability effect, the centrality effect, the endowment effect, and the less is more effect. For these phenomena, we examined whether a model’s mechanism could provide an explanation. Some other phenomena are not readily simulated with certain models. For example, the MDFT and MLBA models do not provide a mechanism to simulate the incidental value effect, the attribute distribution effect, the attribute range effect, the attribute spacing effect, and the background contrast effect. We consider the MDFT and MLBA models to be unable to explain these phenomena, unless an explanation was discussed in previous studies.

For the MDFT model, the parameter values we tested are all of the combinations of the followings: ϕ_1_ = [0.01, 0.02, 0.03, . . . , 0.10]; ϕ_2_ = [0.05, 0.06, 0.07, . . . , 0.10]; ξ = [1, 3, 5, . . . , 19]; σ^2^ = [0.01, 0.02, 0.03, . . . , 0.10]; and *T* = [10, 20, 30, . . . , 100]. When testing the effects of time pressure, we multiplied *T* with 2 to examine whether an effect becomes stronger.

For the MLBA model, the parameter values we tested are all of the combinations of the followings: *m* = [0, 2, 4, . . . , 100]; λ_1_ = [0.0, 0.2, 0.4, . . . , 10.0]; λ_2_ = [0.0, 0.2, 0.4, . . . , 10.0]; and *I*_0_ = [0, 2, 4, . . . , 100].

For the CCM model, the parameter values we tested are all of the combinations of the followings: θ = [0.0, 0.1, 0.2, . . . , 10.0] and λ = [1.00, 1.01, 1.02, . . . , 10.00].
